# c-MYC overexpression induces choroid plexus papillomas through a T-cell mediated inflammatory mechanism

**DOI:** 10.1186/s40478-019-0739-x

**Published:** 2019-05-29

**Authors:** Ashirwad Merve, Xinyu Zhang, Nicola Pomella, Serena Acquati, Joerg D. Hoeck, Anaelle Dumas, Gabriel Rosser, Yichen Li, Jennie Jeyapalan, Silvia Vicenzi, Qianhai Fan, Zeng Jie Yang, Arianna Sabò, Denise Sheer, Axel Behrens, Silvia Marino

**Affiliations:** 1 Blizard Institute, Barts and The London School of Medicine and Dentistry, Queen Mary University of London, 4 Newark Street, London, E1 2AT UK; 2 Adult Stem Cell Laboratory, The Francis Crick Institute, 1 Midland Road, London, NW1 1AT UK; 3 Department of Experimental Oncology, European Institute of Oncology, Via Adamello 16, 20139 Milan, Italy; 4 Fox Chase Cancer Centre, Temple University, Philadelphia, USA

**Keywords:** C-MYC, Mouse models, Choroid plexus tumours, Inflammation

## Abstract

Choroid plexus tumours (CPTs) account for 2–5% of brain tumours in children. They can spread along the neuraxis and can recur after treatment. Little is known about the molecular mechanisms underlying their formation and only few high fidelity mouse models of p53-deficient malignant CPTs are available.

We show here that c-MYC overexpression in the choroid plexus epithelium induces T-cell inflammation-dependent choroid plexus papillomas in a mouse model. We demonstrate that c-MYC is expressed in a substantial proportion of human choroid plexus tumours and that this subgroup of tumours is characterised by an inflammatory transcriptome and significant inflammatory infiltrates. In compound mutant mice, overexpression of c-MYC in an immunodeficient background led to a decreased incidence of CPP and reduced tumour bulk. Finally, reduced tumour size was also observed upon T-cell depletion in CPP-bearing mice. Our data raise the possibility that benign choroid plexus tumours expressing c-MYC could be amenable to medical therapy with anti-inflammatory drugs.

**Electronic supplementary material:**

The online version of this article (10.1186/s40478-019-0739-x) contains supplementary material, which is available to authorized users.

## Introduction

Choroid plexus tumours (CPT) are intracranial neoplasms derived from the choroid plexus epithelium. They predominantly occur in children and account for 2–5% of all paediatric brain tumours, with relatively higher incidence in infants [1]. They mainly occur in the ventricles and mostly present with severe hydrocephalus. They may spread along the neuraxis and can recur after treatment. Histologically CPTs are classified into three categories - Choroid Plexus Papilloma (CPP, benign, World Health Organisation [WHO] grade I), Atypical Choroid Plexus Papilloma (ACPP, intermediate, WHO grade II) and Choroid Plexus Carcinoma (CPC, malignant, WHO grade III) [46]. CPC is known to be associated with younger age at diagnosis as compared to CPP [23].

Limited literature is available on the molecular mechanisms mediating the biology of these tumours. Merino et al. demonstrated that CPPs and ACPPs are molecularly distinct from CPCs at the genetic, epigenetic and expression level but molecular similarities suggest CPPs and ACPPs are a single entity [28]. In keeping with this interpretation another study showed that CPP and ACPP have similar cytogenetic features and that ACPP represent an immature variant CPP characterised by increased proliferative activity, whereas CPCs represent a genetically distinct group [18]. Tumour aggressiveness and poorer survival outcome in CPTs is associated with a greater number of copies of mutated *TP53* [28, 44] and CPCs are also found in patients with Li-Fraumeni syndrome (germline mutation of *TP53*) [52] . Methylation profiling applied to CPTs led to the identification of 3 distinct subgroups with clinical relevance and confirmed that CPCs cluster separately from the majority of CPPs and ACPPs [46].

Current management for choroid plexus tumours includes initial surgery with an aim to achieve gross total resection (GTR) which is associated with decreased risk of recurrence. Achieving GTR can be challenging (achieved in only approximately 63–70% of CPTs) [8] and, as for all neurosurgical procedures, can be associated with postoperative co-morbidities such as visual changes (up to 16%) and seizures (up to 13%) [1]. CPCs have a 20 fold higher risk of metastasis and local recurrence as compared to CPP [52]. Initial resection is followed in these cases by adjuvant therapy (radiotherapy and/or chemotherapy). Currently there are no disease-specific chemotherapeutic agents available. Indeed identifying druggable molecular aberrations for these tumours has been challenging, possibly because of their rarity and paucity of faithful pre-clinical experimental models. In particular, mouse models of CPPs are lacking and high fidelity models only exist for CPCs occurring in a Tp53-deficient background [10, 47].

The MYC family members, c-MYC, N-MYC and L-MYC, are well characterised regulators of cellular processes such as proliferation, apoptosis and cellular metabolism [22, 39]. Their essential role in embryonic stem (ES) cells and in early embryonic mouse development has long been known [6]. The key role of c-MYC in stem cell biology has been strengthened by the observation that its expression together with OCT4, SOX2 and KLF4 is sufficient to revert terminally differentiated fibroblasts into a pluripotent embryonic stem cell-like state [45]. *c-MYC* is also one of the first proto-oncogenes identified, it is overexpressed in a large number of tumours and its active role in oncogenic transformation of normal cells is well documented [43]. In brain tumourigenesis, deregulation of the expression of c-MYC has been shown to be functionally relevant for medulloblastoma and glioblastoma, the most common intrinsic brain tumours in children and adult respectively (reviewed in [9]). *MYC* genes, most commonly *c-MYC* and *MYCN*, are frequently amplified in medulloblastoma [35] and are associated with a poor prognosis [38] and/or tumour recurrence [15]. In mouse models, inactivation of Tp53 and Pten in adult NSPCs induces high grade glial tumours through increased cellular c-Myc activity which leads to impaired differentiation, enhanced self-renewal capacity of NSPCs and tumour-initiating cells (TIC) as well as maintenance of TIC tumourigenic potential [53]. Moreover, human glioblastoma initiating cells preferentially express c-MYC and depend on its activity for self-renewal and tumour growth [48] and recently a novel MYC-mediated metabolic reprogramming has been described in these cells [49].

Here, we set out to analyse the impact of genetically engineered c-MYC overexpression in NSPCs on brain tumourigenesis in the mouse.

## Results

### Generation of a mouse line which overexpresses c-MYC in a spatiotemporally regulated manner

The Gateway Entry system [32] was chosen to guarantee controlled and efficient monosite insertion of the human *c-MYC* construct into the ubiquitously expressed ROSA26 locus [32] (Additional file 1: Figure S1A). This approach was used to ensure the overexpression achieved would be low level, thus mimicking the degree of c-MYC overexpression observed in human malignancies (reviewed in [30]). Genotyping of the chimeras was performed using primers to detect the eGFP reporter gene (Additional file 1: Figure S1B), germline transmission and line establishment was achieved (*STOPFloxc-MYC*). Expression of human c-MYC was confirmed by western blot analysis in NSPCs isolated from the postnatal transgenic mouse SVZ and treated with A-Cre (Additional file 1: Figure S1C). Concomitant expression of GFP was seen in these cultures (Additional file 1: Figure S1D), as expected because of the *IRES-eGFP* sequence contained in the targeting construct (Additional file 1: Figure S1A).

The mouse line generated here allows for low level c-MYC overexpression in the desired cell population at the desired time.

### Choroid plexus tumours developed at high penetrance in NestinCre;STOPFlox-c-MYC transgenic mice

**Fig. 1:**
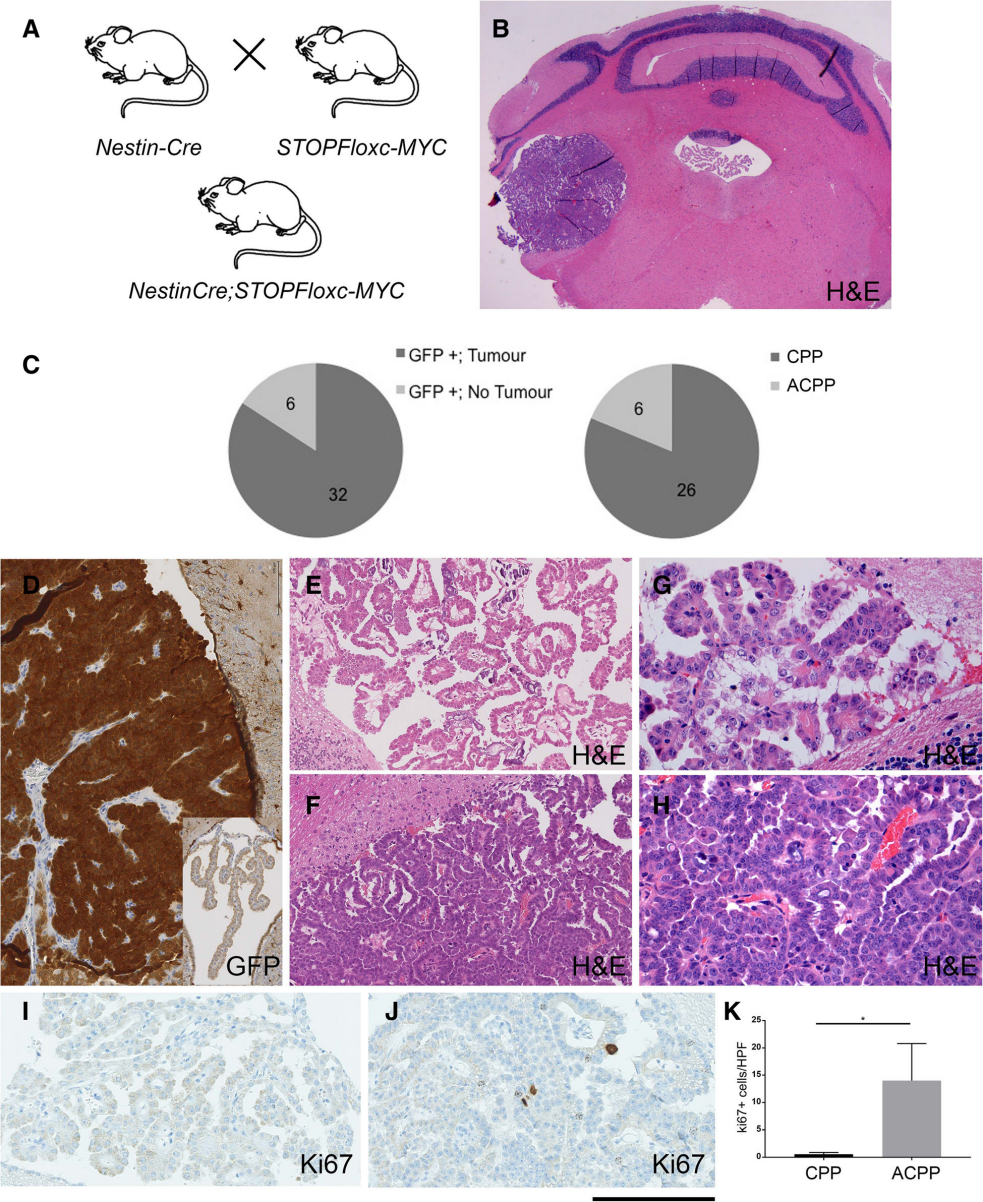
CPT develop at high penetrance in NestinCre;STOPFloxc-MYC mice*.* **a** Schematic of generation of double transgenic *NestinCre;STOPfloxc-MYC* mice. **b** CPT arising in a double transgenic mouse, with a tumour seen within the lateral ventricle (left) as compared to normal choroid plexus in the fourth ventricle (centre). **c** A large proportion (84.2%) of compound mice developed CPT (pie graph on the left); the majority of them (81.3%) were CPP, whist the rest (18.8%) were ACPP, (graph on the right). **d** GFP immunohistochemistry showing strong and even expression in the CPT as compared to control CP (inset). **e**-**h** H&E of CPT. CPP shows mild stratification of cells albeit with largely preserved papillary architecture and with mild cellular and nuclear pleomorphism (**e** low magnification; **g** high magnification) while ACPP showed moderate cellular and nuclear pleomorphism with more blunting of papillary architecture (**f** low magnification and **h** high magnification). In addition ACPPs showed significantly higher Ki-67 labelling as compared to CPPs (**i** CPP; **j** ACPP). **k** Ki67 quantification (Bar graph representing average cells/HPF as mean ± SEM, *n* = 3 in each cohort, * *P* < 0.05). Scale bar = 1 mm (**b**), 250 μm (**d**, **e**, **f**) and 125 μm (**g**, **h**, **i**, **j**). Abbreviations: CP – choroid plexus; CPT-choroid plexus tumour; ACPP – atypical choroid plexus tumour; CPP – choroid plexus papilloma; NSPCs – neural stem progenitor cells; H&E – haematoxylin and eosin; GFP – Green Fluorescent Protein, indicative of c-MYC expression

To model c-MYC induced brain tumourigenesis, *STOPFlox-c-MYC* mice were crossed with *NestinCre* mice (Fig. 1a), a Cre driver known to target NSPCs and the progeny derived thereof from E10.5 onwards [7]. The offspring were crossed between each other to obtain *NestinCre;STOPFloxc-MYChet* (heterozygous *c-MYC* allele), *NestinCre;STOPFloxc-Mychom* (homozygous *c-MYC* allele) and single transgenic littermates as controls. We included in the experimental setting both heterozygous and homozygous transgenic mice to also assess the impact of c-MYC dosage on NSPCs functional properties in vivo.

Fifty-one double transgenic mice (43 *NestinCre; STOPFloxc-MYChet* and 8 *NestinCre; STOPFloxc-MYChom*) were generated and kept under tumour watch; mice were killed when symptomatic or at termination of the experiment (20 months).

Post mortem examination of the brains of these animals revealed choroid plexus tumours (CPT) in a high proportion of the mutant mice (c-MYC^Over^ CPT) (Fig. 1b, c). Immunohistochemistry for GFP confirmed activation of the construct in the choroid plexus of 38/51 mutant mice with equal distribution between heterozygous and homozygous genotypes (Fig. 1d). CPT were found in 32 of these mice (84.2% penetrance) (Fig. 1c) and occurred in both genotypes (the penetrance in heterozygous and homozygous was 87.9 and 60% respectively; the difference was not statistically significant) (Additional file 2: Table S1).Histologically the tumours showed papillary architecture with epithelial multilayering and focal solid pattern of growth, mild to moderate cellular and nuclear pleomorphism, in some cases with prominent nucleoli. Subarachnoidal spreading and/or brain invasion was noted in a proportion of the cases. Those tumours with mild cellular stratification, preserved papillary architecture, mild cellular and nuclear atypia and with mitoses of 2 or less/10 HPF were regarded as choroid plexus papilloma (CPP, Fig. 1e and g). Those tumours with patchy solid growth, more pronounced (moderate) atypia with prominent nucleoli and presence of mitotic activity of 3 or more/10 HPF were classified as atypical choroid plexus papilloma (ACPP, Fig. 1f and h). ACPPs had significantly higher Ki-67 proliferation index as compared to CPP – mean values of 14.0 vs 0.55 Ki67+ cells/HPF respectively (*n* = 3, *p* = 0.04, Fig. 1i-k). Of the 32 tumours, 26 (81.3%) were CPP (Fig. 1c) and 6 (18.7%) were ACPP (Fig. 1c), 29 were in heterozygous and 3 were in homozygous compound mutant. Of the 6 ACPPs, 2/3 were in homozygous compound mice and 4/29 in heterozygous compound mice. Six of the thirty two mice with CPT developed symptoms between 11 and 18 months. Severe hydrocephalus was detected in 6/32 (18.8%) mice with CPTs, among which 4 (66.6%) showed ACPP histology. The CPTs were marginally more frequently detected in the fourth ventricle (in 28 of 32, 87.5%) as compared to lateral ventricle (75%), whilst atypia was more common in the fourth ventricle (5 of 6, 83.3%). Only 1 of 32 tumours (CPP) showed dystrophic calcification. Severe pleomorphic features and necrosis were not seen. A retrospective analysis showed that tumours developed as early as 5 months of age. The tumour characteristics are summarised in Additional file 2: Table S1.

No CPT were detected in 34 control mice (*STOPFloxc-MYC*, single transgenic) and the choroid plexus of these mice were negative for GFP. No other brain tumours were detected in the double transgenic mice.

### c-MYC overexpression in the epithelial lining of the postnatal CP induces neoplastic transformation

Analysis of the Allen Brain Atlas spatial transcriptomic datasets revealed robust expression of c-Myc in the CP of adult mice, whilst no expression was noted at embryonic timepoints (Additional file 3: Figure S2A-F). Meta-analysis of human and murine expression datasets confirmed that both species expressed similar levels of c-MYC (Additional file 3: Figure S2H), there was good concordance of gene expression (Additional file 3: Figure S2I), the majority of expressed genes were shared (Additional file 3: Figure S2J) and these genes belong to key molecular pathways (Additional file 3: Figure S2K), hence supporting further investigation of this model of c-MYC tumourigenesis.

**Fig. 2:**
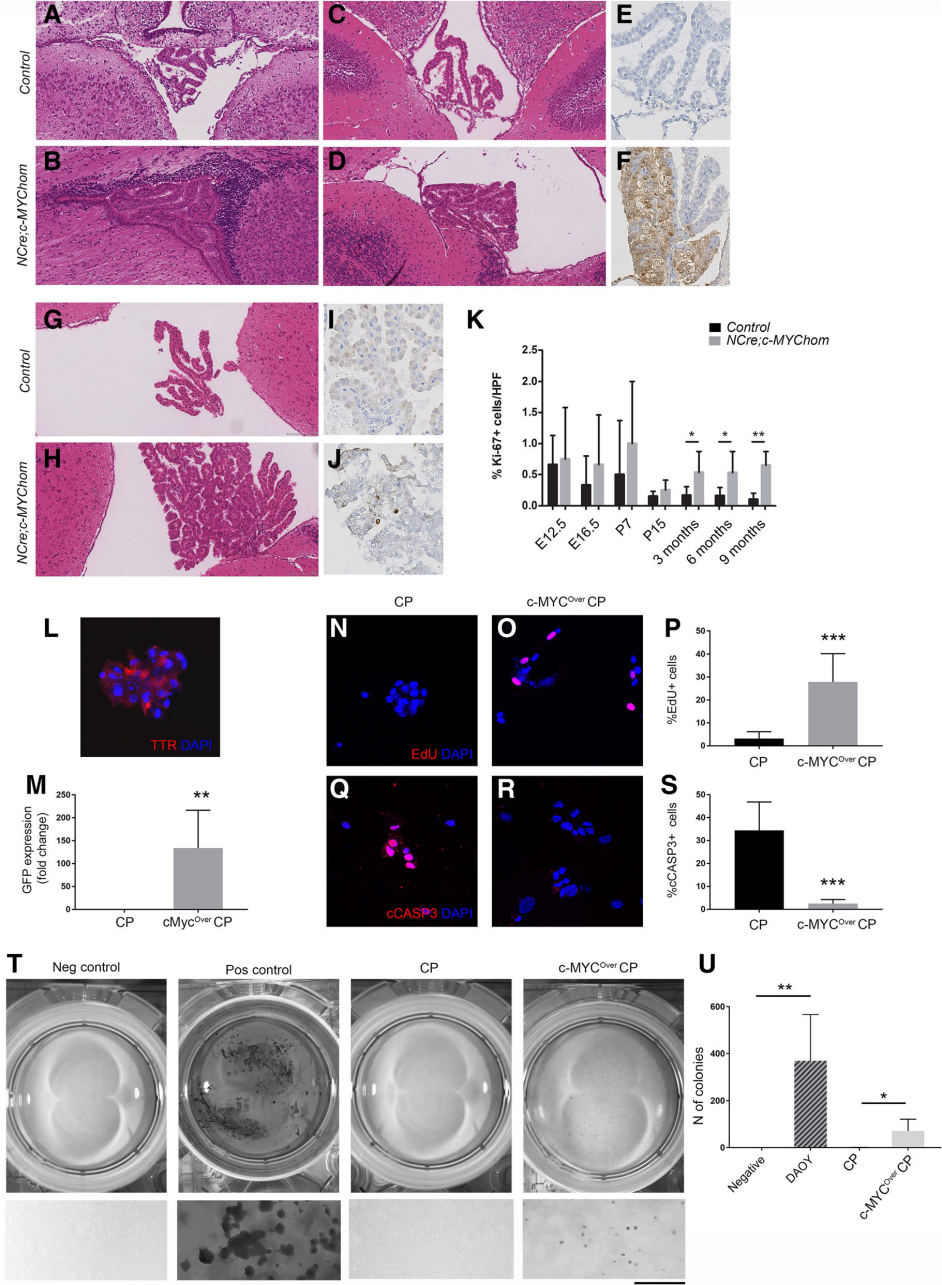
Increased proliferation and decreased apoptosis in CP cells overexpressing c-MYC. **a**, **b** Slightly enlarged CP was observed in *NestinCre;STOPfloxc-MYC* mice at P7 as compared to controls. No histological atypia was noted. **c**-**f** Patchy hyperplasia with mild nuclear atypia was seen in *NestinCre;STOPfloxc-MYC* mice at P15 in comparison to controls. The hyperplastic areas showed GFP positivity in *NestinCre;STOPfloxc-MYC* mice, in keeping with activation of c-MYC construct. **g**, **h** At 3 months, widespread CP hyperplasia with mild nuclear atypia was observed in c-MYC overexpressing mice. **i**-**k** A trend toward increased proliferation was observed in the CP of *NestinCre;STOPfloxc-MYC* mice at E12.5, E16.5, P7 and P15, a finding which reached statistical significance from 3 months onwards (*n* = 6 in each cohort for 3 months and 6 months; *n* = 4 in each cohort for 9 months). **l** Immunolabelling for transthyretin (TTR) confirmed the epithelial nature of the majority of the cultured cells. **m** GFP expression upon transduction with adeno-Cre (A-Cre) confirmed construct activation. **n**-**p** Increased proliferation on Edu immunoassay and **q**-**s** decreased apoptosis on cCasp3 immunoassay upon c-MYC overexpression by A-Cre induced construct activation (cMYC^Over^CP) as compared to control CP cells. **t** Soft agar colony formation assay demonstrating cMYC^over^CP cultures forming colonies as compared to control CP. A negative (no cells) and positive control (DAOY, medulloblastoma cell line) are included on the left; bottom panel shows high magnification images. **t** Quantification of the finding (*n* = 3). **k**, **m**, **p**, **s**, **u**– Bar graph representing Mean ± SEM; * *P* < 0.05; ** *P* < 0.01; *** *P* < 0.001. Scale bar is 125 μm (**l**, **n**, **o**, **q**, **r**)

**Fig. 3:**
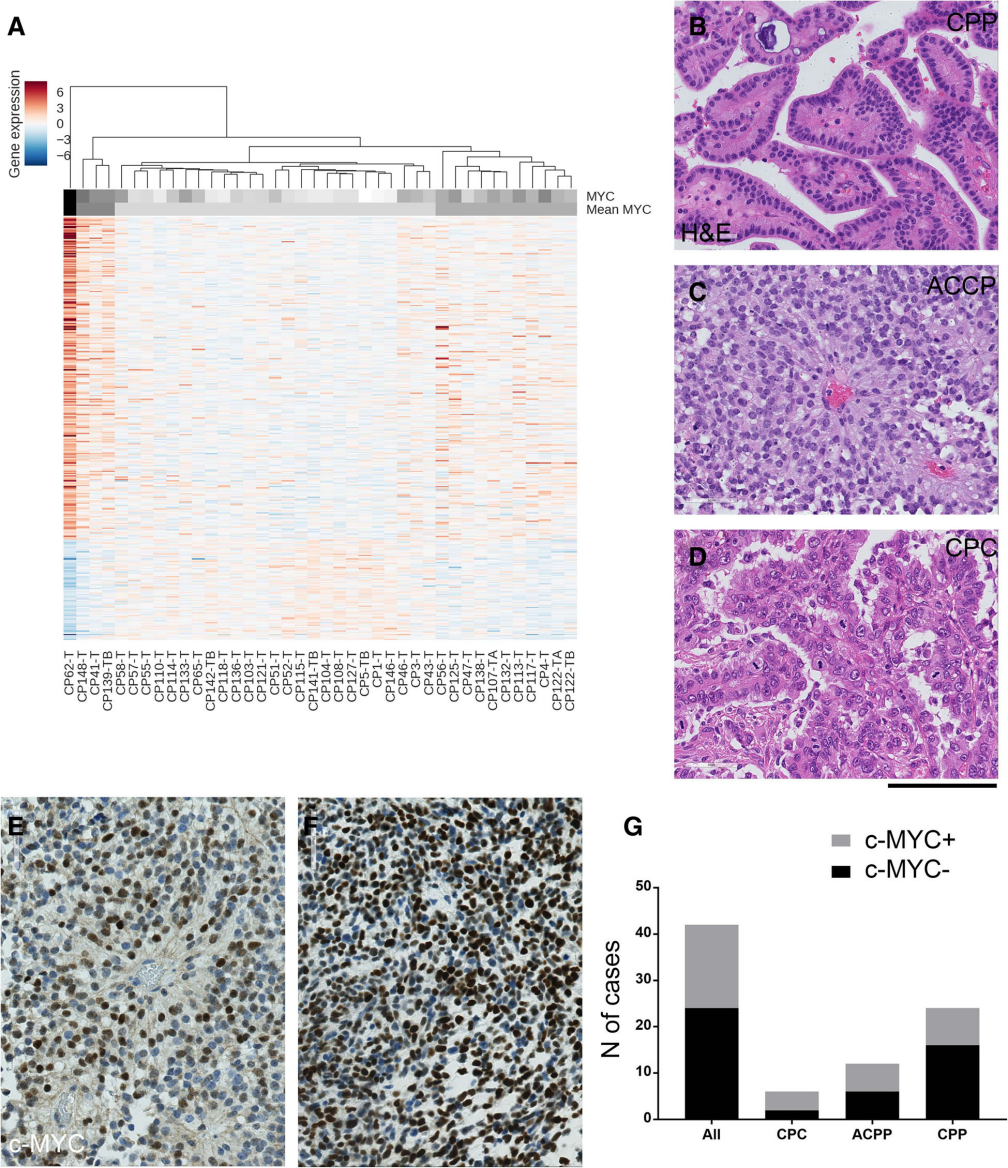
c-MYC is expressed in a proportion of human CPTs*.* **a** Unsupervised hierarchical clustering analysis of 356 genes, the expression of which significantly correlated with *c-MYC* expression in 40 CPTs from Merino et al. dataset, showing clustering of CPTs based on c-MYC expression. The heat map colour for each tumour is defined by the Pearson correlation coefficient between the log-ratio expression profiles of genes. **b**-**g** Representative histology of the analysis of an independent (Brain UK) cohort of human CPT: papilloma, WHO grade I (**b** CPP), atypical papilloma WHO grade II (**c** ACPP) and carcinoma, WHO grade III (**d** CPC). **e**-**f** c-MYC immunohistochemistry showing mostly diffuse expression (**e**) and in some cases focal expression (**f**). Nuclear expression of c-MYC in more than 10% was considered as positive. **g** Graphical representation of cases positive for c-MYC, overall and among different histological subtypes. 18/42 (43%) of all cases were c-MYC positive; 4/6 (67%) CPC, 6/12 (50%) ACPP and 8/24 (33%) CPP were c-MYC positive. Scale bar = 125 μm (**b**, **c**, **d**, **e**, **f**)

Firstly, we set out to define the biological history of these tumours. Histological analysis of *NestinCre; STOPFloxc-MYC* double transgenic brains at embryonic and postnatal timepoints (E12.5, E16.5, P7, P15, 3 months) showed no abnormalities at E12.5 and E16.5 and no expression of GFP (data not shown). The CP was slightly more bulky at P7 (Fig. 2a, b), although no definite atypia was seen and a staining for GFP was negative (data not shown). Patchy hyperplasia with mild nuclear atypia was noted at P15 (Fig. 2c, d), a timepoint where expression of GFP in the hyperplastic areas was noted (Fig. 2e, f) in keeping with activation of the construct in the epithelial lining. Similar findings were found at 3 months (Fig. 3g, h), when significantly increased proliferation was first noted (Fig. 2i-k).

Interestingly, at 5–6 months of age morphological abnormalities in keeping with CPP were detected in 3 of 6 (50%) compound mutant mice (2 het, 1 hom), with cellular stratification, multi-layering and mild nuclear atypia of the epithelium. CPP were detected in 3 of 4 mice (75%, 2 het 1 hom) at 9–12 months of age (Additional file 2: Table S1). Increased proliferation was observed in compound mutant mice – 0.53 vs 0.16 (*p* = 0.034) and 0.65 vs 0.1 (*p* = 0.005) - at 5–6 months and 9–12 months respectively (Fig. 2k). There was no significant difference in apoptosis as assessed by immunohistochemistry for cCASP3, a central executor in the main apoptotic pathway, at all time points analysed (data not shown).

The co-expression of CFP and transthyretin in scattered epithelial cells of the postnatal mouse CP in Nestin-CFP mice [12] suggested that our construct was expressed in the epithelial lining of the CP (Additional file 3: Figure S2G). To confirm that it was c-MYC overexpression in the CP epithelium what led to tumour formation, we set up to isolate, culture and functionally analyse primary CP cells from newborn *STOPFlox-c-MYChom* mice and confirmed in the first instance that they were composed of epithelial CP cells by means of immunostaining for transthyretin (over 95% of the cell population was TTR+, Fig. 2l). A-Cre infection induced recombination of the construct and its concomitant activation was confirmed by assessment of GFP expression in the culture (c-MYC^Over^ CP) (Fig. 2m). Increased proliferation, as assessed by Edu immunolabelling (Fig. 2n, o-p) and decreased apoptosis, as determined by cCASP3 staining (Fig. 2q-s, *n* > 3), was observed upon c-MYC overexpression. A soft agar colony formation assay confirmed that epithelial CP cells overexpressing c-MYC acquired anchorage independent growth (Fig. 2t, u), providing evidence of cellular transformation in vitro [16].

In summary, these experiments show that low level overexpression of c-MYC in the CP epithelium induces CPP with high penetrance.

### c-MYC is expressed in a substantial proportion of human choroid plexus tumours

Next, we set out to interrogate a publicly available gene expression dataset [Affymetrix Human Exon 1.0 ST platform, GEO GSE60886] [28] of 40 human CPTs to assess whether expression of c-MYC would characterise a proportion of these tumours and whether genes could be identified linked to c-MYC expression. We show that c-MYC is expressed in a proportion of CPTs and we identify 356 genes of over 18,000 genes targeted by the core probes on the exon array, the expression of which was significantly correlated with c-MYC expression (absolute Pearson correlation coefficient *r* ≥ 0.5, *p* < 0.01). Hierarchical clustering of the samples based on the expression levels of these genes showed partition into high, medium and low c-MYC expressing tumours (Fig. 3a).

Next, we assembled an independent cohort of 42 CPTs comprising 24 CPPs, 12 ACPPs and 6 CPC to validate c-MYC expression (Additional file 4: Table S2). Histology was reviewed and both diagnosis and grading confirmed (Fig. 3b-d). Immunohistochemistry was performed to determine c-MYC expression and tumours were classified as either positive or negative. While most of the positive tumours showed focal/patchy expression (Fig. 3e), two of them showed diffuse expression (Fig. 3f). A total of 18/42 (43%) tumour cases showed positive immunolabelling for c-MYC, among which 4/6 (67%) CPC, 6/12 (50%) ACPP and 8/24 (33%) CPP were positive (Fig. 3g). There was no significant correlation between c-MYC expression and tumour grading or between c-MYC expression and age of the patients (data not shown). Evidence of *c-MYC* amplification at the DNA level was not found in any of the tumour samples expressing c-MYC, as assessed by FISH (data not shown), in keeping with lack of significant correlation between c-MYC expression and gain of Chromosome 8 as found in the Merino et al. dataset. No correlation was found between TP53 staining and c-MYC expression (data not shown), a finding which was also in keeping with the results of our analysis of the Merino et al. dataset.

These results show that a proportion of CPT expresses c-MYC in two large cohorts of these tumours.

### Deregulation of inflammatory pathways in c-MYC overexpressing CPT

Next, we looked more closely at the list of genes significantly correlated with c-MYC expression in human CPT and noticed that 11 out of the top 20 positively correlated genes were inflammation related, mostly chemokines and their receptors (Additional file 5: Figure S3A). Functional annotation clustering of all 356 genes using Ingenuity® Pathway Analysis confirmed that the majority of canonical pathways significantly enriched in CPT in this gene list were immune related (Additional file 5: Figure S3B, C and D).

**Fig. 4:**
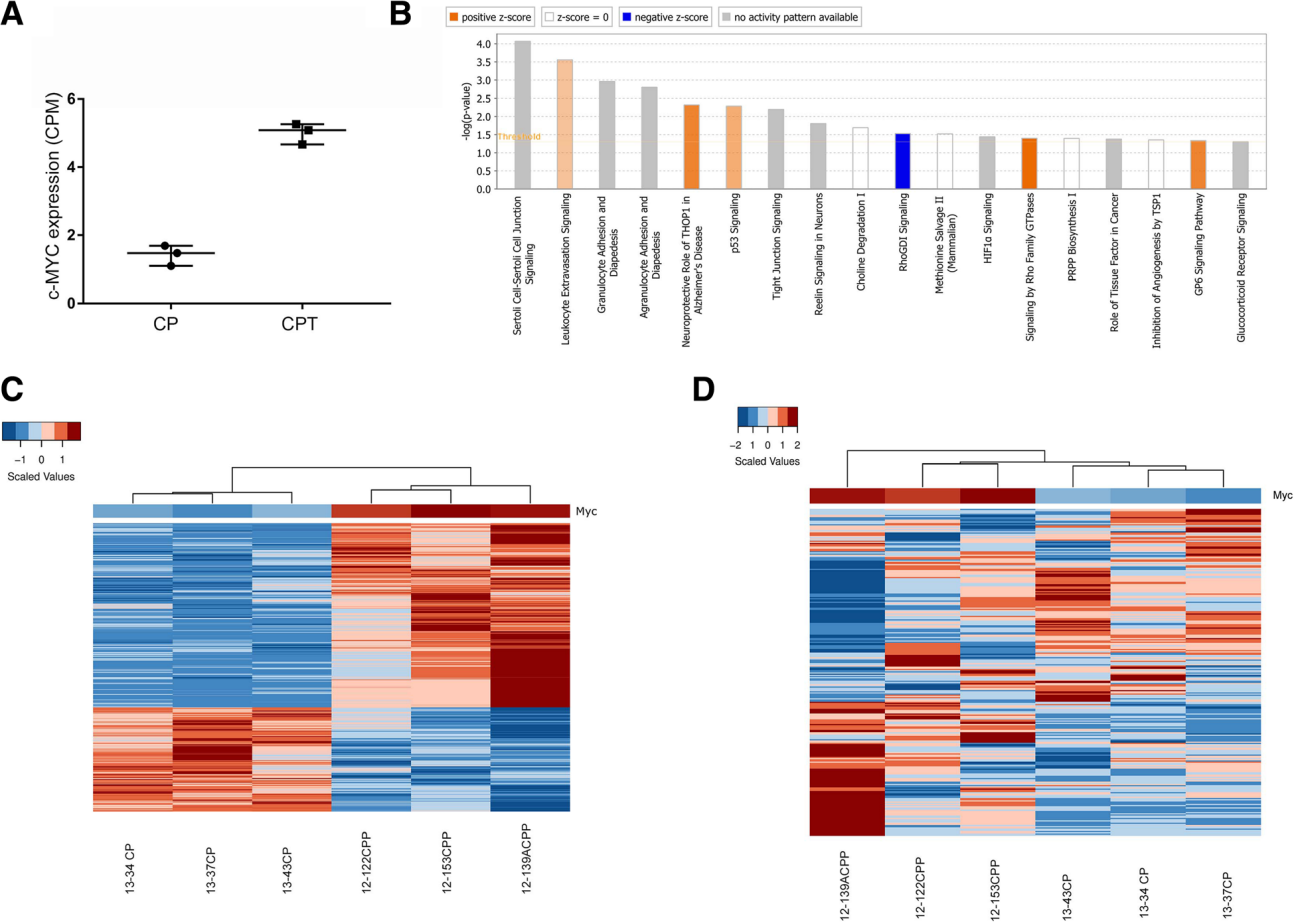
Deregulation of inflammatory pathways in NestinCre;STOPfloxc-MYC CPT. **a** Normalized c-MYC expression values (CPM) of 3 murine control and 3 CPT samples. Median and interquartile range are depicted. **b** IPA analysis on differentially expressed genes (*n* = 245) between murine CPT and control samples. Positive z-score is associated with enrichment in the CPT context. **c** Unsupervised hierarchical clustering analysis and relative expression of c-MYC-correlated genes in the murine context (*n* = 2290), across control and CPT samples. **d** Unsupervised hierarchical clustering analysis and relative expression of murine orthologs of *c-MYC*-correlated genes in the human context (*n* = 356), across control and CPT samples

To assess whether the transcriptional signature in our murine model would recapitulate the observation in human c-MYC+ CPT, we performed differential expression analysis between 3 murine CPT and 3 control CP samples. Significant overexpression of c-MYC was confirmed in the CPT samples (Fig. 4a) and expression of c-MYC targets, belonging to a cell-type independent c-MYC signature [19], was found enriched in CPT relative to control mice (Additional file 6: Figure S4A).

Two hundred forty-five genes were significantly deregulated (FDR < 0.05) in murine CPT and the enrichment of pathways such as Leucocyte extravasation signalling, Granulocyte/Agranulocyte adhesion and diapedesis, Tp53 signalling pathways, among others, was seen in CPT context (Fig. 4b).

Next, we performed a correlation analysis (absolute Pearson coefficient *r* ≥ 0.5, *p*-value < 0.05) and 2290 genes were retained, the expression of which significantly correlated with c-MYC expression in murine CPT samples. The corresponding heatmap shows a clear partition between control and CPT samples (Fig. 4c). Also, a similar pattern emerged from the expression of the murine orthologs of the 356 c-MYC-correlated genes found in human CPTs (Fig. 4d), confirming activation of the MYC signalling cascade in human c-MYC+ tumours and murine CPT.

**Fig. 5:**
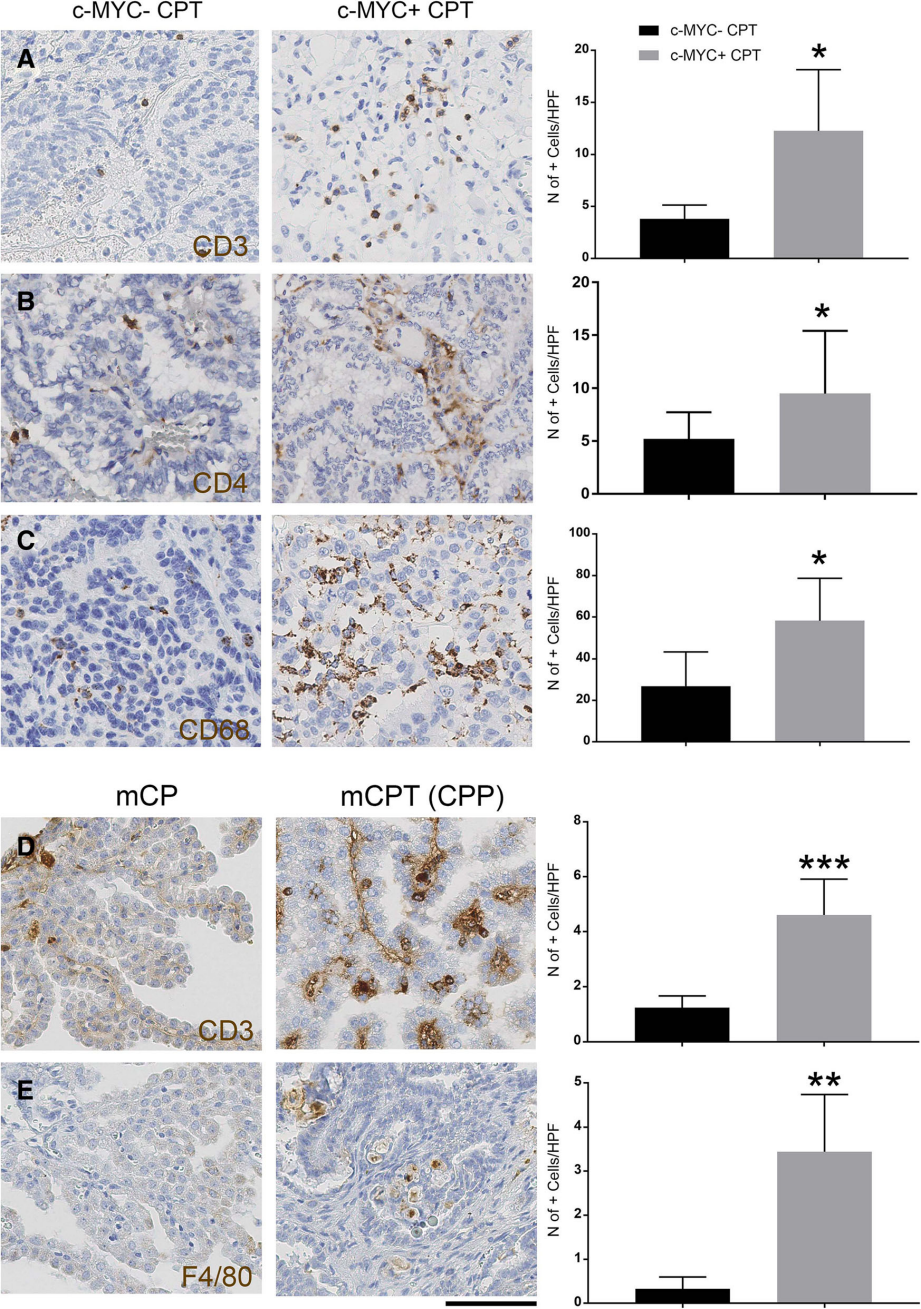
Characterisation of the inflammation in c-MYC+ CPT and c-MYC^Over^ CPT*.* **a** An increased T-lymphocytic infiltrate on CD3 immunohistochemistry in c-MYC+ tumours in relation to c-MYC- is noted (quantification showed on bar graph on the right; Mean ± SEM; *n* = 4 in each cohort; * *P* < 0.05). **b** Subtyping of T-lymphocytes showed predominant increase in CD4 subtype population (quantification showed on bar graph on the right; Mean ± SEM; *n* = 11 for c-MYC+ and *n* = 10 for c-MYC-; * *P* < 0.05). **c** Pronounced macrophage infiltration was also observed among c-MYC+ on CD68 immunohistochemistry (quantification showed on bar graph on the right; Mean ± SEM; *n* = 4 in each cohort; * *P* < 0.05). **d**, **e** Comparative analysis of the CPT (CPP) developing in the *c-MYC* overexpressing mouse model as compared to normal CP showed T-lymphocytic (**d**) and macrophagic (**e**) infiltration in the tumour parenchyma. Quantification is shown on bar graph on the right; **d** Mean ± SEM; *n* = 6 in CPT and *n* = 7 in control; *** *P* < 0.001 and **e** Mean ± SEM; *n* = 7 in CPT and *n* = 3 in control; ** *P* < 0.01. Scale bar = 125 μm (**a**, **b**, **c**, **d**, **e**)

To assess whether these transcriptomic data reflected a differential inflammatory cell recruitment to c-MYC expressing CPT, we stained our cohort of human CPT tumour samples for CD3 (T-lymphocyte marker) and CD68 (macrophage marker) and comparatively quantified the number of positive cells in c-MYC+ and c-MYC- tumours. We show a significantly increased number of T-lymphocytes within the c-MYC+ tumours as compared to c-MYC- tumours − 12.28 vs 3.8 CD3+ cells/HPF (Fig. 5a, *p* = 0.046). Quantification of T-lymphocyte subtypes by CD4 and CD8 immunostaining revealed that it was the CD4+ component which was responsible of the observed increase of T-lymphocytes - 9.49 vs 5.2 CD4+ cells/HPF between c-MYC+ tumours as compared to c-MYC- tumours (Fig. 5b, *p* = 0.027). Whilst the number of CD3+ cells was higher than the one of the CD4+ cells, as predicted, in the c-MYC+ tumours − 12.28 CD3+ cells and 9.49 CD4+ cells- this was not the case in c-MYC- tumours − 3.8 and 5.2- and the reason for this discrepancy is at present unclear. Nevertheless a convincing higher number of CD3+ and CD4+ cells were found in c-MYC+ tumours as compared to c-MYC- tumours, which is well in keeping with the results of the transcriptomic analysis.

A significantly increased number of macrophages was also observed in c-MYC+ tumours as compared to c-MYC- tumours - 58.25 vs 26.87 CD68+ cells/HPF (Fig. 5c, *p* = 0.03). There was no significant difference in CD8+ subpopulation among these two cohorts (Additional file 7: Figure S5A) and no significant difference in B-lymphocyte infiltrates, as assessed by CD20 immunostaining, was observed (Additional file 7: Figure S5B).

Comparative analysis of the murine CPT versus normal CP confirmed a significant number of T-lymphocytes and macrophage in the tumours – 4.61 vs 1.24 CD3+ cells/HPF for T-lymphocytes (Fig. 5d, *p* = 0.00075) and 3.44 vs 0.33 F4–80+ cells/HPF for macrophages (Fig. 5e, *p* = 0.011). Analysis of earlier time points in *NestinCre;STOPFlox-c-MYC* revealed no statistically significant increase in infiltrating T-lymphocyte or macrophage in the choroid plexus of double transgenic mice as compared to wild-type mice until 9 months (Additional file 7: Figure S5C, D).

These finding show a significant inflammatory component in the human CPT expressing c-MYC, which was recapitulated also in the murine model generated by genetically engineered *c-MYC* overexpression in the CP epithelium.

### Crossing onto a NOD-SCID background and CD3 depletion in vivo reduces tumour bulk in NestinCre; STOPFlox-c-MYC mice

**Fig. 6:**
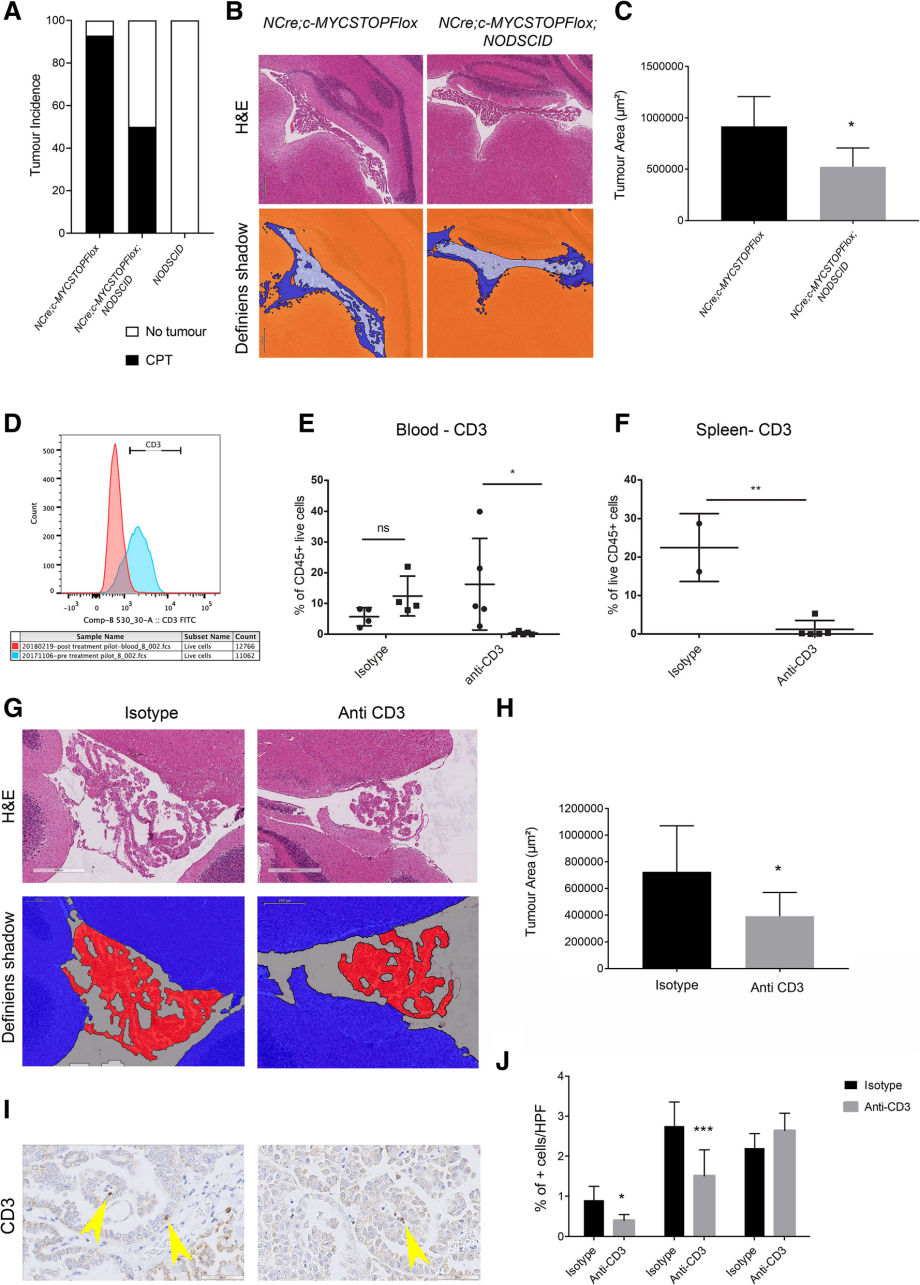
CD3 depletion in NestinCre; STOPFloxc-MYC mice reduces proliferation and bulk of CPT*.* **a** A significant reduction in tumour incidence was noted in the triple compound mutant (*NestinCre; STOPFlox-c-MYC;NOD-SCID*) as compared to double compound mutant (*NestinCre; STOPFlox-c-MYC*). NODSCID mouse group confirmed no tumour development without c-MYC overexpression in an immunocompromised background. **b**, **c** Tumour area reduction, as assessed by Definiens® image analysis software in the triple compound mutant (**b** right) as compared to double compound mutant (**b** left). Representative tumour histology (**b** upper panel) and tumour area measured on Definiens (**b** lower panel, no colour). **c** Quantification bar graph with mean area ± SEM; *n* = 6 for double transgenic and *n* = 5 for triple transgenic; * *P* < 0.05. **d** Flow peaks showing reduction in CD3+ cells in blood following anti-CD3 injection (pre-treatment blue, post treatment red peaks). **e** Reduction in CD3+ population was noted post injection in the anti-CD3 treated group in comparison to pre injections. No significant changes where observed in the isotype treated group. Common Leukocyte Antigen - CD45+ CD3+ cells were selected; Circles = pre-injection levels, Squares = post injection levels after 4 weeks, each representing one mouse. Mean ± SEM represented; * *P* < 0.05. **f** Similar results were observed in spleen where a significant reduction of CD3+ cells was noted in post injection anti-CD3 cohort in comparison to isotype control; Mean ± SEM represented; ** *P* < 0.01. **g** H&E of CPT assessed by Definiens® image analysis software showed a reduced tumour area in the anti-CD3 injected cohort in comparison to isotype injected tumours. Representative tumour histology (**g**, upper panel) and tumour area measured on Definiens (G, lower panel, red) and quantification in (**h**) bar graph with mean area ± SEM; *n* = 9 for anti-CD3 treated and *n* = 8 for isotype control; * *P* < 0.05. **i**, **j** Reduced number of CD3+ cells (arrow head) was observed in anti-CD3 treated group (right panel) in comparison to isotype control (left panel). **j** Quantification bar graph of Ki67+ and CD3+ cells with mean cells/HPF ± SEM; *n* = 9 for anti-CD3 treated and *n* = 8 for isotype control; * *P* < 0.05; *** *P* < 0.001. Scale bar = 400 (G top) 200 μm (**g** bottom) and 50 μm (**i**)

To assess the functional role of the inflammatory infiltrates in the initiation of tumourigenesis in murine CPT, we crossed *NestinCre; STOPFlox-c-MYC* mice onto a *NOD-SCID* background. We show a significant reduction in tumour incidence in the triple compound mutant (*NestinCre; STOPFlox-c-MYC;NOD-SCID*) as compared to double compound mutant (*NestinCre; STOPFlox-c-MYC*) generated from the same crossing (50% vs 92.8%, *p* < 0.05) (Fig. 6a, *n* = 14 for double transgenic and *n* = 8 for triple transgenic). In addition, the size of the tumour, as assessed by Definiens digital tissue image analysis software on serial sections, was smaller in the triple mutant mice as compared to the double mutant mice (Fig. 6b, c, *n* = 6 for double transgenic and *n* = 5 for triple transgenic cohorts).

Next, we set out to systemically deplete T cells in tumour bearing *NestinCre; STOPFloxc-MYC* mice to assess the impact of inflammation on tumour growth in a setting that would more closely mimic a therapeutic approach. A T cell depletion strategy was chosen because of the increase in CD3+ T-lymphocyte in murine CPT and the enrichment for T-lymphocyte-regulatory pathways in the geneset significantly correlated with c-MYC expression in human CPT.

Effective suppression of CD3+ cells in the blood and in the spleen of mice treated with anti-CD3 antibody was shown as compared to mice injected with isotype control (Fig. 6d-f). Further subtyping of the CD3+ cells revealed that the depletion strategy mainly impacted the CD4+ T cell fraction whilst no significant effect was seen on the CD8+ fraction (Additional file 8: Figure S6A-C). Double labelling also revealed that it was the CD4+ and FoxP3+ fraction of T-cells which were particularly impacted by the treatment (Additional file 8: Figure S6D). Significant reduction of the tumour area was found in the anti-CD3 injected cohort (Fig. 6g, h, *n* = 9 for anti-CD3 treated cohort and *n* = 8 for isotype control cohort) as assessed by Definiens digital tissue image analysis software on serial sections encompassing the entire tumour area. Immunohistochemical analysis confirmed reduction of CD3+ positive T cells (Fig. 6i, j, *n* = 9 for anti-CD3 treated cohort and *n* = 8 for isotype control cohort). No significant impact of the CD3 depletion strategy was noted on macrophage infiltrates (Fig. 6j).

We conclude that T-lymphocyte depletion in murine c-MYC driven CPT has an anti-tumourigenic effect.

## Discussion

We show here that c-MYC overexpression in the choroid plexus epithelium induces inflammation-dependent choroid plexus papillomas in a mouse model. c-MYC is expressed in a proportion of human choroid plexus tumours and this subgroup of tumours is associated with an inflammatory transcriptome and significant inflammatory infiltrates.

The expression of c-MYC in neural progenitor cells (NSPCs) was achieved via Nestin-driven Cre expression. The line is known to enable widespread recombination of LoxP flanked constructs in the developing CNS, including the CP as described in the original characterisation of the expression pattern of the line in a crossing with a reporter mouse [7]. Nestin has been shown to be expressed in the roof plate, an organising centre from which CP epithelial cells originate [2]; however we do not find evidence of construct activation during embryonic development. CP epithelial cells have been shown to upregulate the expression of Nestin under specific conditions, such as the exposure to CSF obtained from patients with acute traumatic brain injuries [13]. In fact we found that activation of the construct in the CP occurred early postnatally, reflecting a physiological expression pattern for Nestin.

CPTs developed with high penetrance (84.2%) in these mice from 5 months of age.

The majority of the tumours observed fulfilled the WHO 2017 criteria for a diagnosis of CPP, however nearly a fifth of them displayed atypical features, in keeping with ACPP. Definite malignant features which would be required for the diagnosis of CPC were not found in any of the tumours examined. c-Myc overexpression in combination with Tp53 deficiency, has been shown to induce aggressive CPC at 100% penetrance in less than 150 days in the mouse [10]. Alterations in cell cycle regulation and DNA damage responses were shown at transcriptome level raising the possibility that these molecular mechanisms could be pathogenetically relevant. Deregulation of mechanisms involved in DNA maintenance and repair had been previously linked to CPC pathogenesis (38% penetration in less than 220 days) in a mouse model where deletion of *Tp53*, *Rb* and *Pten* was achieved in newborn mice by electroporation of Cre Recombinase into the hindbrain CP epithelium at E12.5 [47]. Comparison of differentially expressed transcriptome signatures of both models revealed a 60% overlap with an enrichment of signalling pathways involved in cell proliferation and DNA damage responses [10], in keeping with the shared concomitant Tp53 deficiency.

We describe here the first mouse model of CPP/ACPP in a genetic background retaining expression of Tp53 (Additional file 6: Figure S4B). We show expression of c-MYC in a proportion of human CPTs in two large cohorts as well as a lack of association of c-MYC expression and *TP53* mutations/deficiency in both cohorts. Therefore, our model recapitulates the phenotype/genotype of a subgroup of benign human CPT. Overexpression of c-MYC alone in NSPCs was recently reported to induce CPCs at full penetrance at 8 weeks of age with tumours displaying a full blown malignant phenotype, including mitotic activity above 5/10HPF, severe nuclear pleomorphism and necrosis [41]; increased ribosome biogenesis was suggested as potential pathogenetic mechanism in these tumours [41]. We never observed CPCs in our compound mice, in keeping with previous reports in both mice [10, 47] and human [52] of *TP53* deletion/inactivating mutations underlying the vast majority of CPC. It is conceivable that differences in expression levels of c-MYC as well the different Cre driver used could explain this difference, although it is unclear which human CPC, the Shannon et al. model would recapitulate.

Increased proliferation and reduced apoptosis were observed in c-MYC^Over^ CP cells and increased colony formation in the soft agar assay confirmed the transformation potential of the genetic modification introduced. These in vitro results were paralleled by modest increased proliferation in vivo, which was not counteracted by increased apoptosis both in vitro and in vivo. Overexpression of c-MYC led to the formation of CPT but it was not sufficient to elicit the formation of neural tumours, the latter finding confirming previous reports [34]. These results are in keeping with a model whereby increased expression levels of c-MYC elicit different biological outputs in a tissue-specific fashion and is in keeping with previous studies [29].

Interestingly, meta-analysis of a publicly available transcriptomic dataset of human CPTs [28] showed expression of c-MYC in a proportion of tumours. Expression of c-MYC in human CPTs of all grades was confirmed in a large independent cohort of these tumours, which we assembled in the UK. We found that gene amplification at the DNA level was not the cause of c-MYC expression, a finding in agreement with an indirect oncogenic activation of c-MYC. Deregulation of c-MYC expression is observed in more than half of human cancers and can be a consequence of gene amplification, chromosomal translocation, and/or protein stabilization [17]. Most frequently though, oncogenic activation of c-MYC is indirect, for example due to dysregulation of pathways physiologically regulating c-MYC (reviewed in [22]). In CNS tumours, c-MYC overexpression without underlying gene amplification is seen in glioblastoma [4] and in the WNT subgroup of medulloblastoma [31].

The CP is functionally responsible for maintaining the blood-CSF barrier and contributes to mounting a cellular response to infection via production of cytokines and chemokines such as interleukins, TNF-α attracting inflammatory cells [reviewed in [40]. Perhaps unsurprisingly then, an inflammatory signature has been described in a proportion of benign CPT [14, 33]. We show here that it is linked to c-MYC expression.

Interestingly, we found a highly significant correlation of the expression of inflammatory cytokines and their receptors with c-MYC expression in the Merino et al. transcriptomic dataset of human CPTs and in our mouse model. Importantly, the number of CD3+ T-lymphocytes (with predominantly CD4+ T-helper cell population) and CD68+ macrophages infiltrating the c-MYC+ CPTs of our cohort was higher as compared to c-MYC- CPTs. Because inflammatory infiltrates with a similar cell composition were observed in our c-MYC-driven mouse model, we reasoned that the inflammatory component was likely induced by c-MYC. Because no inflammation was noted in other c-MYC-driven CPT models [10, 41], it is conceivable that lack of concomitant inactivation of *Tp53* could contribute to this phenotype.

Links between tumours and inflammation are well established, although the molecular mechanisms underpinning these links are not clearly understood and are very diverse in different tumour types (reviewed in [11]). Two main pathways define the tumour/inflammation connection- an extrinsic pathway, driven primarily by inflammatory conditions which increase oncogenic risk (such as inflammatory bowel disease causing bowel cancer); and an intrinsic pathway, driven by genetic alterations such as oncogenic mutation in *RAS*, *c-MYC* and *RET* causing activation of downstream inflammatory signalling (reviewed in [25]). c-MYC is overexpressed in many tumours, where its role in regulating cellular proliferation and survival is well known (reviewed in [5]). c-MYC function in remodelling the extracellular microenvironment via regulation of anti-tumour immunity has also been shown [3] and c-MYC-regulated inflammatory responses to tumourigenesis have been characterised [21, 51].

The significantly lower incidence of CPP in an immunodeficient background (NOD-SCID) together with the reduced tumour mass in those compound mutant mice developing tumours supports a pro-tumour role of the inflammation in our experimental setting. Reduction of the CPP tumour mass upon treatment with a T-cells depletion therapy mainly impacting on the CD4+ fraction of T-cells and not impacting on the macrophages, confirmed the finding in a setting more closely mimicking a potential therapeutic approach. Whether the macrophage infiltration observed could contribute to the phenotype in addition to the T-cell infiltration is at present unclear.

A recent randomised, double-blind, placebo-controlled trial shows the impact of an anti-inflammatory therapy with canakinumab targeting the IL-1β innate immunity pathway on lung cancer incidence and mortality [36]. Our findings in benign CPP/ACPP provide the molecular foundation for further investigation of the potential role of anti-inflammatory drugs, in particular IL-1 receptor antagonists such as Anakinra (IL-1Ra) or Canakinumab (human monoclonal antibody specifically targeting IL-1b) in the treatment of c-MYC+ choroid plexus tumours and raise the possibility that the spectrum of tumours which may be responsive to anti-inflammatory therapy could be broader than currently thought.

## Material and methods

### Generation of a c-MYC overexpressing mouse line

The Gateway Entry system was chosen to guarantee controlled and efficient monosite insertion of the *c-MYC* construct into the ubiquitously expressed *ROSA26* locus [32]. The toxin-encoding *ccdB* gene was excised from the pENTR1A vector (Invitrogen) by BamH1/Xho1 restriction digest and a 1.5 kb cDNA construct of human *c-MYC* was inserted into these sites of the pENTR1A vector. BamH1/Xho1 restriction digest showed correct insertion of the construct. Correct orientation of the insert was confirmed by DNA sequencing (data not shown). The *c-MYC* construct was subsequently inserted into a targeting vector via in vitro recombination. Since the *c-MYC* construct in the pENTR1A vector is flanked by specific lambda phage integrase recognition sites (attL), the *c-MYC* construct could be efficiently transferred to a targeting vector carrying the corresponding heterotypic sites (attR). The targeting vector contains a 5′ homology region to the mouse *ROSA26* genomic locus, a splice acceptor (*SA*) site, a *PGK-neo-3x pA* stop cassette flanked by *loxP*-sites (LSL), the *c-MYC* construct, an *IRES-eGFP* reporter gene, a 3′ homology region to the mouse *ROSA26* genomic sequence and a *Diphteria Toxin A (DTA) selection cassette* (Additional file 1: Figure S1A). It was electroporated into G4 F1 hybrid ES cells and screening for positive clones with correct insertion was performed by PCR using a forward primer in the genomic *ROSA26* locus 5′ of the targeting vector and a reverse primer in the 5′ region of the targeting vector (Additional file 1: Figure S1A). Correctly targeted clones showed a 1.3 kb band as a result of the PCR screening. This was further validated by Southern Blot analysis (data not shown). Three of the positive ES cell clones (1B12, 2D4 and 2B8) were selected for injection into blastocysts from C57BL/6 mice to generate chimeric mice. This part of the procedure was performed by the London Research Institute Transgenic Service. Genotyping of the chimeras was performed using primers to detect the *eGFP* reporter gene. Germline transmission and line establishment was achieved for clone 1B12, from now on referred to as *STOPFloxc-MYC*.

### Mouse strains, genotyping procedures and construct activation

A *NestinCre* transgenic line [7] was used to activate c-MYC expression from the *STOPFloxc-MYC* construct and *NODSCID* mice were used to generate the triple compound mutant mice. Ear notches of transgenic mice were digested in lysis buffer (50 mM Tris, pH 8.0, 100 mM EDTA, 100 mM NaCl and 1% SDS) and Proteinase K (Biolabs) for 3 h at 55^o^ C. DNA was precipitated in isopropanol-2 (Fisher Scientific) and dissolved in TE buffer (10 mM Tris,1 mM EDTA, pH 8.0) for 30 min at 55^o^ C. Genotypes of mice were determined by PCR, using published primer sequences [26, 27, 50]. All procedures had Home Office approval (Animals Scientific Procedures Act 1986, PPL 70/6452).

### Choroid plexus cultures

CP tissue was dissected from brain ventricles of neonatal *STOPFloxc-MYC* mice aged 3–5 days postpartum. The tissue was washed with Hanks’ Balanced Salt solution (HBSS, Sigma) and digested in pre-warmed and freshly prepared pronase/HBSS solution (2 mg/ml in HBSS and filtered through a 0.22 μm filter unit) at 37 °C for 5 min. The reaction was stopped by adding complete growth medium (Dulbecco’s modified Eagle’s medium nutrient mix F-12 supplemented with 10% fetal bovine serum, 50 U/ml penicillin, 50 μg/ml streptoMYCin, 2.5 μg/ml amphotericin). The digested tissue was collected by centrifugation at 1000 g for 2 min. The pellet was re-suspended in complete growth medium by gentle pipetting up and down (15–20 times) to generate a suspension of single cells and cell aggregates. The CP cells were then counted and seeded on the culture plate for growing. Twenty μM of Ara-C was added into the cells overnight to inhibit the fibroblast cells proliferation. Medium was changed every 2 days until the cells reached confluence for passaging. After passaging, either Cre or GFP adenovirus was added to activate the construct.

### Patients and tumour samples

Paraffin embedded tissue blocks or glass slides of CPT samples were obtained from Oxford University Hospitals NHS Trust, Nottingham University Hospitals NHS Trust, Imperial College Healthcare NHS Trust and University College London Hospitals NHS Trust under the UK Brain Archive Information Network (BRAIN UK) umbrella (Approval no. 18/001 incorporating previous 13/003 and 15/007). Samples were also received from Children’s Cancer and Leukaemia Group (CCLG) University of Leicester (CCLG Biological Studies Steering Group Approval no. 2013 BS 03). Informed consent was obtained from all of the patients or parents/guardians in case of children. Ethical Approval from UK Research Ethics Committee was available under the BRAIN UK and CCLG umbrella. The tissue samples were received, stored and processed as per protocol.

We studied 42 tumour samples from 40 patients (adults and children) including recurrence tumour samples from 2 of the adult patients (Additional file 4: Table S2). Among the recurrences, histological grading remained the same in one, whereas there was histological progression in the other. Histopathological review was carried out by two board certified neuropathologists (AM and SM). Immunohistochemical analysis for INI1 (hHF5) was performed on all cases to exclude atypical teratoid/rhabdoid tumour (AT/RT) [20] and cases with INI1 mutation (negative staining) were excluded from the study.

### Immunohistochemistry and immunocytochemistry

Formalin fixed paraffin embedded (FFPE) tissue sections of mouse brain and human CPT samples were used for immunohistochemistry. The mouse samples were processed at UCL IQ path laboratory and the human samples at the Histopathology Department, The Royal London Hospital, Great Ormond Street Hospital and UCL IQ path. Dewaxing, antigen retrieval and pre-treatment with appropriate serum was performed as per published protocols. The following primary antibodies were used for mouse tissue on automated Ventana Discovery XT platform: Monoclonal antibody for the green fluorescent protein (GFP) from the jellyfish *Aequorea Victoria* (Abcam, ab290) 1:1500, CD3 (Leica/Novocastra, LN10) 1:100, F4-80 (Abcam, ab6640) 1:100, Ki-67 (Cell Signalling, 12202S) 1:100 and Caspase-3. (Cell Signalling, 9661 L) 1:100. Anti-rabbit (Dako, E0353), anti-rat (Dako, E0468) and anti-mouse (Dako, E0354) secondary antibodies were used as required. For human CPTs the following primary antibodies were used on Launch 16,000 Optimax autostainer: C-MYC (Abcam, ab32072) 1:50, CD3 (Leica, NCL-L-CD3-565) 1:50, CD4 (SP35, Roche), CD8 (SP57, Roche), CD20 (L26, Roche) prediliuted, CD68 clone KP1 (Dako, M0814) 1:150. P53 staining was performed using clone D07 (Ventana, 05278775001) prediluted on Ventana Ultra autostainer and INI1 (BD Biosciences, 612110)) 1:100 using BondMax automated stainer. Anti-mouse or anti-rabbit secondary antibodies were used as appropriate.

Immunocytochemistry was carried out on cells plated on poly-L-Lysine (Sigma) coated glass coverslips. The cells were washed briefly in PBS and then fixed in 4% paraformaldehyde solution for 15 min. Blocking and staining were carried out according to standard protocols. Antibodies: anti-transthyretin (1:500, Abcam), anti-cleaved caspase 3 (1:500, Abcam) in 200 μl of blocking buffer and incubated for 3 h at room temperature. Secondary antibodies applied to the cells: donkey polyclonal anti-rabbit IgG-546 (1:500, ThermoFisher) or donkey polyclonal anti-sheep IgG-594(1:250, Abcam) in 200 μl blocking buffer and incubated in the dark for 1 h at room temperature.

For the EdU staining, Click-iT™ EdU Alexa Fluor™ Imaging Kit (ThermoFisher) was used. Cells were pre-treated with 10 μM of EdU for 3 h at 37 °C before fixation. After washing with PBS, cells were permeabilized with 0.1% Triton X-100 (Sigma) for 20 min at room temperature. The Click-iT reaction cocktail was freshly prepared according to the instruction. Cells were incubated in this reaction cocktail for 30 min in the dark. After 3 washes (5 min each) in PBS, stained cells were mounted with Mounting Medium with DAPI (Vector Labs) on a glass microscope slide. Cells were viewed by Zeiss 710 Confocal Microscope.

For quantification, 5 and 3 high power fields (representing a 400-fold magnification) were captured for each sample in human and mouse tumours respectively. Positive cells and total number of cells were counted by a researcher blinded to the experimental conditions of each slide.

### Fluorescent in-situ hybridisation (FISH)

Unstained microtome sections of the FFPE tumour samples were analysed for *C-MYC* and *MYCN* amplification by FISH. The procedure was carried out at Camelia Botnar Laboratory, Great Ormond Street Hospital. FFPE sections of 4 μm thickness on glass slides were dried and de-waxed by heating to 60 °C in a dry oven, then washed in Xylene. The xylene was removed by passing the slides through a series of ethanol washes. The slides were then placed in a saline sodium citrate (SSC 2X) solution at 70 °C for 1 h. After washing in distilled water the slides were then digested using a 4 mg Pepsin solution in 0.2 N hydrochloric acid solution at 37 °C for 20 min. The slides were then washed in distilled water and dehydrated by passing through a series of alcohol washes. FISH probe sets for *MYC/CEP8* and *MYC/CEP8/IGH* (Abbott, USA) and *MYCN/AFF3* (Leica, Germany) were prepared and hybridised according to the manufacturers’ instructions. Cell images were captured using Olympus BX61 (Olympus, Japan) and Zeiss Axioskop Imager 1 (Zeiss, Germany) microscopes; image analysis was performed using Cytovision (Leica, UK), Isis (Metasystems, Germany) and SmartCapture (Digital Scientific, UK) software. For analysis, a target number of 100 representative informative cells were examined from each hybridisation, with 50 cells being scored independently by two analysts.

### Gene expression profiling and pathway analysis

Gene expression data measured on the Affymetrix Human Exon 1.0 ST array platform were obtained from Gene Expression Omnibus (GEO, accession GSE60892) for 40 patient tumour samples [28]. Six probes targeted c-MYC, five of which were positively correlated, with all pairwise Pearson correlation coefficients (r) greater than 0.5: 3115514, 3115515, 3115522, 3115523 and 3115524. To identify genes that correlated with c-MYC expression, Pearson correlation was performed on all probes against each of these five probes. We identified 1652 probes showing an absolute *r* ≥ 0.5 and *p*-value < 0.01 with at least one of the five c-MYC probes. The normalized expression values were standardised across each of these probes by subtracting the median value and dividing by the interquartile range. We then computed the mean standardised value of each of the genes represented across the 1652 probe list. Comparing each of these aggregated genes against *c-MYC*, we retained 356 genes with absolute *r* ≥ 0.5 and *p*-value < 0.01 (Supplementary Material Genelists). A similar analysis was performed on the RNASeq dataset of 3 murine CPTs, to find genes correlating with *c-MYC* at the expression level, and 2290 genes were retained, with an absolute Pearson coefficient *r* ≥ 0.5 and *p*-value < 0.05.

The differential expression analysis between 3 murine control and 3 CPT samples was performed on edgeR [37], after TMM normalization, with a GLM model. Minimum log-fold change and FDR cut-off were set at 1 and 0.05, respectively.

Adult human and murine CP transcriptomic profiles were obtained from a total of 18 samples (9 human and 9 murine) to carry out a quantitative inter-species comparison at the gene expression level. The analysis was performed in R (version 3.4.3). The datasets are publicly accessible from GEO GSE82308 (6 murine samples), GSE23714 (3 murine samples), GSE68015 (3 human samples) and GSE110226 (6 human samples). To perform the comparative analysis with data obtained from those different experiments and platforms, expression values were normalized with the YuGene package, which uses a cumulative proportion transform [24]. Only the shared genes between the two murine datasets (*n* = 10,300) and the shared genes between the two human datasets (*n* = 14,320) were used for further analysis. Orthologous genes were assessed via Ensembl BiomaRt [42] and the corresponding Bioconductor package in R.

Hierarchical clustering was performed on genes using the Euclidean distance metric and Ward’s method. Pathway analysis of the genes was performed using Ingenuity® Pathway Analysis (QIAGEN Inc., https://www.qiagenbioinformatics.com/blog/discovery/publication-roundup-ingenuity-pathway-analysis-3/).

### Soft agar assay

Cells (7500 per well) were mixed with 0.3% noble agarose in complete growth medium as described above, plated on top of a solid field layer of 0.6% noble agarose in complete growth medium, in a 6-well plate. Cells were fed twice a week with growth medium. After 4 weeks, the colonies were fixed and dyed with Cristal Violet (0.005% in 4% formaldehyde, Sigma), washed with PBS, and imaged. Colonies in the whole plate were then counted and data was analysed by Graphpad Prism.

### CD3 depletion in vivo

*NestinCre;STOPFloxc-MYC* transgenic mice at the age of 9 months were injected with either Hamster anti-mouse CD3 IgG F(ab’)2 clone 145-2C11 (BioXCell Cat no. BE0001-1FAB) or hamster IgG F[ab’]2 isotype control (Cat no. BE0091-FAB) intraperitoneally. Eight mice (all het) were injected with isotype control and 9 mice (2 hom, 3 het) with anti-CD3 antibody at dose of 100 μg/day for 5 days a week, for a duration of 4 weeks. The mice were observed for adverse symptoms and culled at the end of 4 weeks, brains collected, formalin fixed and paraffin embedded.

### Fluorescence-activated cell sorting

*Sampling blood and spleen:* Peripheral blood (~ 30 μl) was collected from tail tip incision prior to injection and post injection by placing the mouse in a strainer. The blood was collected in EDTA coated Eppendorf tubes, treated with lysis buffer and centrifuged and pellet resuspended in FACS buffer. Post injection spleen samples were collected after the mice were culled at the end of 4 weeks observation. The spleen samples were mashed against a cell strainer and washed to break the tissue and collect the spleen cells. Further treatment with lysis buffer and washes were performed before a pellet containing approximately 6 × 10^7^ cells was resuspended for use.

Cells from processed blood and spleen were stained in FACS buffer (1:200 BSA - Sigma, A3912 - and 1:250 EDTA 0.5 mM – Ambion, AM9262 - in PBS). Cells were first treated with Anti-CD16/CD32 FcR blocker (eBiosciences, 140161-82) for 15 min at 4 °C. Antibodies were directly added to blocking solution and incubated for a further 30 min at 4 °C. Cells were then washed with FACS buffer and centrifuged (1500 rpm, 5 min). The cells were re-suspended in fixable fluorescent viability dye diluted in PBS and incubated for 20 min at 4 °C. The cells were then blocked with 4% PFA (1:1 in FACS buffer) for 15 min. For intracellular staining, the cells were stained with antibodies diluted in 70% methanol for 20 min at RT. The fixed cells were stored in FACS buffer at 4 °C. FACS sample were run on an LSR II and were analysed using Flowjo version 10. Following removal of doublets and of dead cells, the gating for markers of interest was done using unstained samples and fluorescent minus one samples (FMOs) as controls. The following fluorochromes were used - CD3-FITC, Fox-P3-PE, CD4-Pacific Blue, CD8-Pe-Cy7, CD45 and F4/80-APC.

### Tumour area assessment

CP and CPT area quantification was carried out on serial sections encompassing the entire CP/tumour with the Definiens Tissue Studio Software. A strategy composed of two steps, tissue detection and ROI detection, was set up to train the software to carry out automatic analysis of all the scanned H&E tissues. A rough tissue detection was first carried out to differentiate tissue from the glass slide, using the threshold pre-defined by the software. Composer initialisation allowed for the selection of subsets (tissue regions selected as examples), necessary for a more detailed definition of tissue versus glass slide. In Composer training, the subsets were segmented (threshold = 6), and tissue areas were selected versus glass slide areas to teach the software. If the learning was adequate it was applied to the whole slide. Under ROI correction, CP and CPT area could be precisely selected. This step was manually carried out for each slide by two researchers blinded to the experimental conditions of each slide.

### Statistical analysis

All quantitative experiments were performed at least in triplicates. A minimum of five high power fields were examined for each sample for each group in human tumours and a minimum of three high power fields in mouse samples, depending on the size of the tumour. Mean values are presented with error bars corresponding to ± SEM. Statistical analysis was performed by using GraphPad PRISM version 7.04 statistical analysis software. Significance is indicated as ∗∗∗*p* < 0.001; ∗∗*p* < 0.01; ∗*p* < 0.05.

## Additional files

Additional file 1: **Figure S1.** Generation of a mouse line to overexpress c-MYC in a spatiotemporally regulated manner. **(A)** Schematic of the Gateway Entry system monosite insertion of the human *c-MYC* construct into the ubiquitously expressed *ROSA26* locus to generate a Cre-activatable *c-MYC* construct. **(B)** Genotyping of the chimeras (het) showing detection of eGFP reporter gene on PCR. Germline transmission and establishment of line *STOPFlox-c-MYC* was achieved. **(C)** Western blot showing expression of human c-MYC in NSPCs isolated from the postnatal transgenic mouse SVZ upon A-Cre infection. **(D)** Immunofluorescence assay showing concomitant expression of GFP in these cultures. Scale bar =125 μm in D. (JPG 530 kb)

Additional file 2: **Table S1.** Characteristics of CPT developing in the transgenic mouse model. Each tumour was histologically classified according to stratification of cells, loss of papillary architecture/solid growth and cellular & nuclear pleomorphism (- mild, moderate or severe). For mitotic activity mild = <2/10 HPF; moderate = 2 to 5/10HPF; severe = >5/10 HPF. Based on the WHO criteria the tumours were classified as either CPP (with mild features) or ACPP (with moderate features). None of the tumours fulfilled the criteria to be classified as CPC. Abbreviations: Het – heterozygous; Hom – heterozygous; Symp – symptomatic; End – end of experiment following 20, 5-6 or 9-12 months of observation (as applicable); F – female; M – male; FV – fourth ventricle; LV – lateral ventricle; CPP – choroid plexus papilloma; ACPP – atypical choroid plexus papilloma; Mod – moderate; Sev – severe. * All mice are of genotype RosaMyc;c-MycSTOPFlox. (DOCX 27 kb)

Additional file 3: **Figure S2.** c-Myc expression pattern in the developing and adult mouse brain. Representative images from Allen Brain Atlas (http://www.brain-map.org/) showing in situ hybridisation (ISH) of c-Myc expression at E18.5 (**A, B, C**) and in the adult brain (**D, E, F**). **G**) Scattered epithelial cells of the postnatal CP express CFP and TTF1 in Nestin-CFP mice. **H**) Normalized c-MYC expression values in adult human and adult murine CP samples (YuGene normalization; median and interquartile range depicted). **I**) Transcriptome-wide Spearman’s rank correlogram of adult human and adult murine samples. The size and the shade of the circles are proportional to the correlation coefficient. MO; Murine, HU; Human. **J**) Venn diagrams showing the number of shared (orthologous) genes between the two species. **K**) Top enriched canonical pathways in human vs. murine CP (IPA, QIAGEN Inc.). Scale bar = 2.5 mm (A, D), 500 μm (B, C, E) and 250 μm (F and G). (JPG 2288 kb)

Additional file 4: **Table S2.** Patient demographics and tumour characteristics. Paediatric and adult patients were included and the tumours samples were of all three histological grades (CPP, ACPP and CPC). Abbreviations: F – female; M – male; FV – fourth ventricle; LV – lateral ventricle; TV – third ventricle; met – metastasis; CPP – choroid plexus papilloma; ACPP – atypical choroid plexus papilloma; IHC – immunohistochemistry. For recurrence, Y = yes; for c-MYC expression, F = focal (10-50%); D = diffuse (>50%). * & ** are tumour tissue from same respective patients. *** is an autopsy case. (DOCX 22 kb)

Additional file 5: **Figure S3.** Inflammatory pathways in c-Myc overexpressing CPT. (A) 11 of top 20 genes significantly correlated with c-MYC expression in CPT from Merino et al. data were inflammation related- mostly chemokines and their receptors; correlation coefficient (r) in relation to c-MYC expression on Y-axis. (B) Pathway analysis of all 356 MYC-correlated genes found in the human context using Ingenuity® Pathway Analysis returned an enrichment of several immune-related canonical pathways. Threshold set at –log (p value) of ≥3. (C and D) Representative immune related pathways from the above analysis are shown; (C) Toll-like receptor signalling pathway and (D) Interleukin-10 (IL-10) signalling pathway; with common elements in the pathway with list of 356 correlated genes in grey/pink. (JPG 773 kb)

Additional file 6: **Figure S4.** Transcriptome analysis of murine CPT. (**A**) Unsupervised hierarchical clustering analysis and relative expression of genes associated with Ji’s signature (*c-Myc* targets) in murine control and CPT samples. (**B**) Expression levels of *Tp53* are not reduced rather increased, albeit not significantly in CPT. (JPG 294 kb)

Additional file 7: **Figure S5.** Inflammatory cells in c-MYC overexpressing human CPT. **(A)** CD8 (cytotoxic T-lymphocyte, a subtype of T-cells) immunohistochemistry shows no significant difference in infiltrate cell counts between c-MYC+ and c-MYC-tumours; quantification shown in bar graph on right hand side; Mean ± SEM; *n* = 10 for c-MYC+ and *n* = 9 for c-MYC- cohorts; ns – no statistical significance. **(B)** CD20 (B-lymphocyte marker) immunohistochemistry shows no significant difference in infiltrate cell counts between c-MYC+ and c-MYC- tumours; quantification shown in bar graph on right; Mean ± SEM; *n* = 11 for c-MYC+ and *n* = 9 for c-MYC- cohorts; ns – no statistical significance. (**C, D**) CD3 and F4-80 immunohistochemistry in *NestinCre;c-MycSTOPFlox* mice at different ages revealed no statistically significant increase in the number of T-lymphocyte (**C**) or macrophage (**D**) in the CP as compared to wild-type mice until tumour development at 9 months of age. Quantification bar graph on right side of C & D, Mean ± SEM; n for CD3 and F4-80 studies in time points P15 = 4, 3m = 3, 6m = 6, 9m = 4 for both mCP and mCPT cohorts; * *P*<0.05. Scale bar = 125 μm (A, B, C, D). (JPG 1187 kb)

Additional file 8: **Figure S6.** Quantification of inflammatory cells in anti-CD3 treated experimental mice. (A-C) A significant reduction in CD4 but not CD8 subpopulation was noted in mouse blood following anti-CD3 treatment. Flow peaks showing a significant difference (red = post treatment, blue = pre-treatment) for CD4 (A) but not statistically significant difference for CD8 (B), which are quantified in the graph in (C) showing mean values ± SEM, *n* = 5 for isotype and *n* = 4 for anti-CD3 treated cohort, * *P*<0.05. (D) Reduction in CD4+ FoxP3+ (regulatory T-cells) population among CD3 T-cell population was noted following anti-CD3 injection, graph showing mean values ± SEM, *n* = 5 for isotype and *n* = 4 for anti-CD3 treated cohort, * *P*<0.05. (E, F) Anti-CD3 treatment had no effect on macrophage (F4-80) population as shown on flow peak (E) and graph in (F) showing mean values ± SEM, *n* = 5 for isotype and *n* = 4 for anti-CD3 treated cohort, * *P*<0.05. A reduction in F4-80 population was noted in post injection isotype treated group. (G) Reduction of mainly CD4, but not CD8 subpopulation is also seen in spleen; graph showing mean values ± SEM, *n* = 5 for isotype and *n* = 4 for anti-CD3 treated cohort, ** *P*<0.01. (JPG 659 kb)
