# Correction to: c-MYC overexpression induces choroid plexus papillomas through a T-cell mediated inflammatory mechanism

**DOI:** 10.1186/s40478-019-0835-y

**Published:** 2019-11-14

**Authors:** Ashirwad Merve, Xinyu Zhang, Nicola Pomella, Serena Acquati, Joerg D. Hoeck, Anaelle Dumas, Gabriel Rosser, Yichen Li, Jennie Jeyapalan, Silvia Vicenzi, Qianhai Fan, Zeng Jie Yang, Arianna Sabò, Denise Sheer, Axel Behrens, Silvia Marino

**Affiliations:** 1 Blizard Institute, Barts and The London School of Medicine and Dentistry, Queen Mary University of London, 4 Newark Street, London, E1 2AT UK; 2 Adult Stem Cell Laboratory, The Francis Crick Institute, 1 Midland Road, London, NW1 1AT UK; 3 Department of Experimental Oncology, European Institute of Oncology (IEO), IRCCS, Via Adamello 16, 20139 Milan, Italy; 4 Fox Chase Cancer Centre, Temple University, Philadelphia, USA

**Correction to: Acta Neuropathol Commun (2019) 7: 95**

**https://doi.org/10.1186/s40478-019-0739-x**

In the original version of this article [1], there was 1 error in the affiliation of the European Institute of Oncology (affiliation 3). In this correction article the updated affiliation is shown for clarification.

The updated information is shown in bold.

Incorrect affiliation:

- *Department of Experimental Oncology, European Institute of Oncology,* Via *Adamello 16, 20139 Milan, Italy.*

Correct affiliation:

- *Department of Experimental Oncology, European Institute of Oncology* ***(IEO), IRCCS****,* Via *Adamello 16, 20139 Milan, Italy.*
