# Supplementary figures and images for: c-MYC overexpression induces choroid plexus papillomas through a T-cell mediated inflammatory mechanism
Source: Acta Neuropathol Commun. 2019 May 29;7:2. doi: 10.1186/s40478-019-0739-x (PMC6540455; doi:10.1186/s40478-019-0739-x)

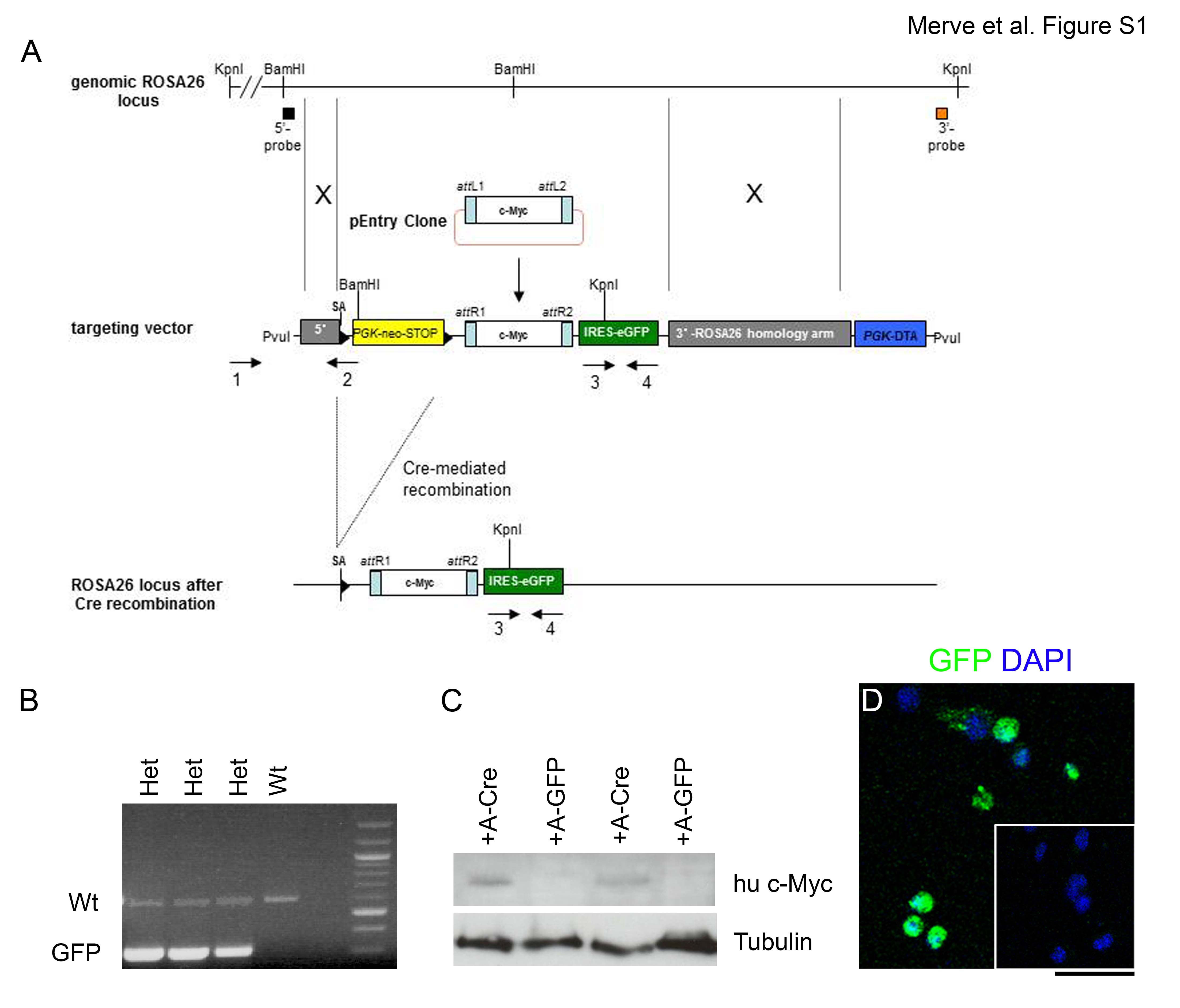

Supplement: Supplementary file 1 — Figure S1. Generation of a mouse line to overexpress c-MYC in a spatiotemporally regulated manner. (A) Schematic of the Gateway Entry system monosite insertion of the human c-MYC construct into the ubiquitously expressed ROSA26 locus to generate a Cre-activatable c-MYC construct. (B) Genotyping of the chimeras (het) showing detection of eGFP reporter gene on PCR. Germline transmission and establishment of line STOPFlox-c-MYC was achieved. (C) Western blot showing expression of human c-MYC in NSPCs isolated from the postnatal transgenic mouse SVZ upon A-Cre infection. (D) Immunofluorescence assay showing concomitant expression of GFP in these cultures. Scale bar =125 μm in D. (JPG 530 kb) [file 40478_2019_739_MOESM1_ESM.jpg]

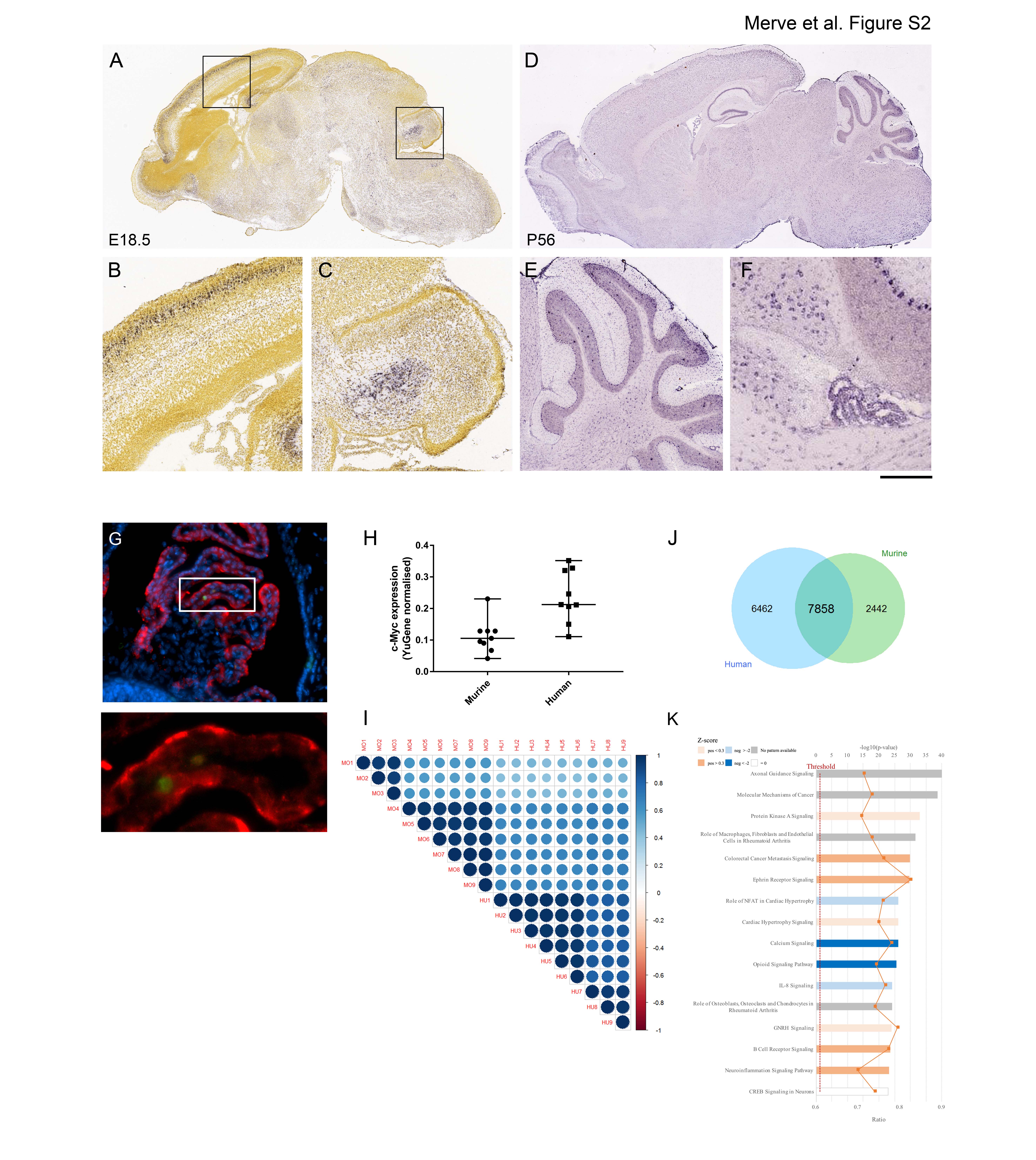

Supplement: Supplementary file 3 — Figure S2. c-Myc expression pattern in the developing and adult mouse brain. Representative images from Allen Brain Atlas (http://www.brain-map.org/) showing in situ hybridisation (ISH) of c-Myc expression at E18.5 (A, B, C) and in the adult brain (D, E, F). G) Scattered epithelial cells of the postnatal CP express CFP and TTF1 in Nestin-CFP mice. H) Normalized c-MYC expression values in adult human and adult murine CP samples (YuGene normalization; median and interquartile range depicted). I) Transcriptome-wide Spearman’s rank correlogram of adult human and adult murine samples. The size and the shade of the circles are proportional to the correlation coefficient. MO; Murine, HU; Human. J) Venn diagrams showing the number of shared (orthologous) genes between the two species. K) Top enriched canonical pathways in human vs. murine CP (IPA, QIAGEN Inc.). Scale bar = 2.5 mm (A, D), 500 μm (B, C, E) and 250 μm (F and G). (JPG 2288 kb) [file 40478_2019_739_MOESM3_ESM.jpg]

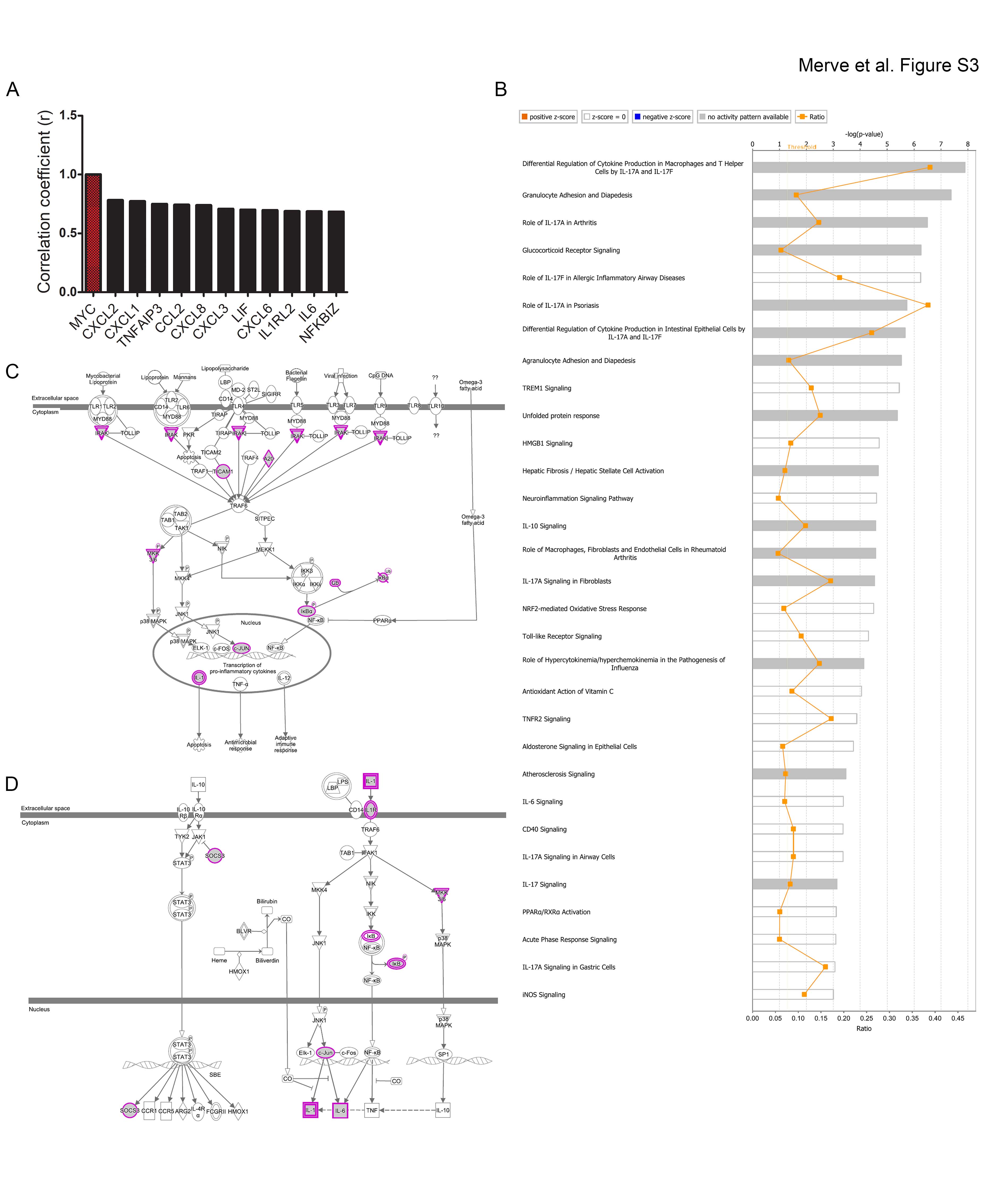

Supplement: Supplementary file 5 — Figure S3. Inflammatory pathways in c-Myc overexpressing CPT. (A) 11 of top 20 genes significantly correlated with c-MYC expression in CPT from Merino et al. data were inflammation related- mostly chemokines and their receptors; correlation coefficient (r) in relation to c-MYC expression on Y-axis. (B) Pathway analysis of all 356 MYC-correlated genes found in the human context using Ingenuity® Pathway Analysis returned an enrichment of several immune-related canonical pathways. Threshold set at –log (p value) of ≥3. (C and D) Representative immune related pathways from the above analysis are shown; (C) Toll-like receptor signalling pathway and (D) Interleukin-10 (IL-10) signalling pathway; with common elements in the pathway with list of 356 correlated genes in grey/pink. (JPG 773 kb) [file 40478_2019_739_MOESM5_ESM.jpg]

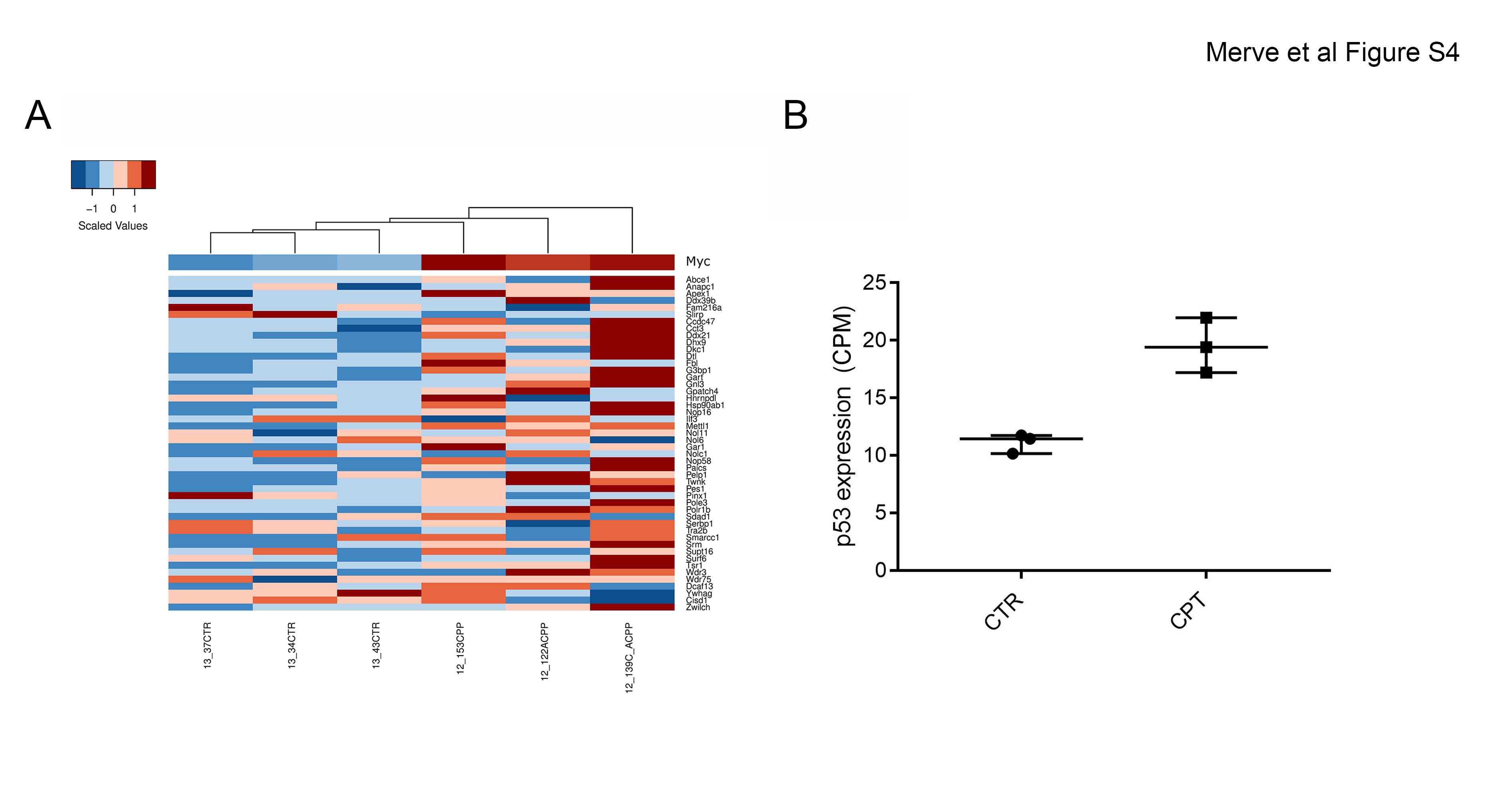

Supplement: Supplementary file 6 — Figure S4. Transcriptome analysis of murine CPT. (A) Unsupervised hierarchical clustering analysis and relative expression of genes associated with Ji’s signature (c-Myc targets) in murine control and CPT samples. (B) Expression levels of Tp53 are not reduced rather increased, albeit not significantly in CPT. (JPG 294 kb) [file 40478_2019_739_MOESM6_ESM.jpg]

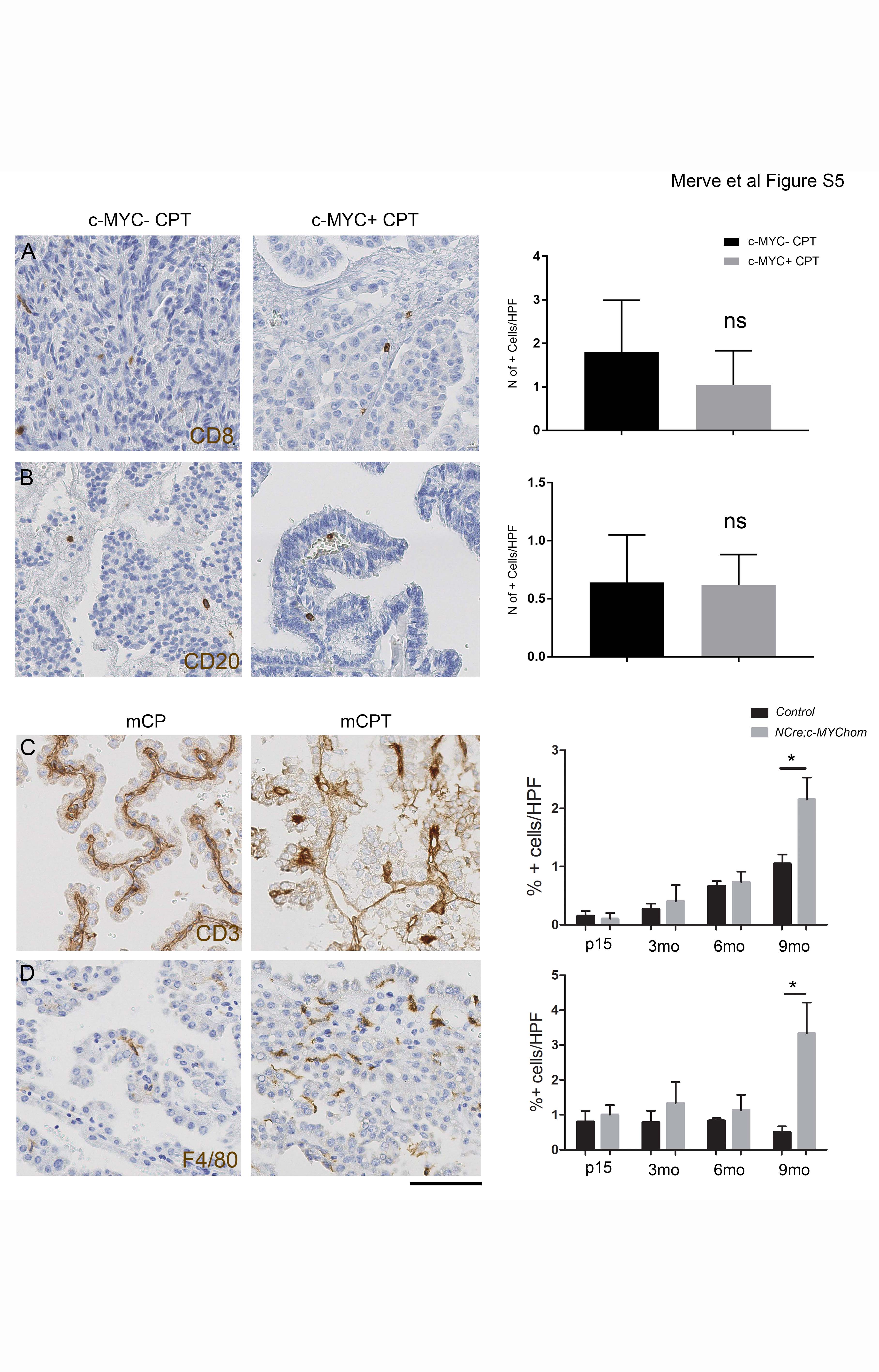

Supplement: Supplementary file 7 — Figure S5. Inflammatory cells in c-MYC overexpressing human CPT. (A) CD8 (cytotoxic T-lymphocyte, a subtype of T-cells) immunohistochemistry shows no significant difference in infiltrate cell counts between c-MYC+ and c-MYC-tumours; quantification shown in bar graph on right hand side; Mean ± SEM; n = 10 for c-MYC+ and n = 9 for c-MYC- cohorts; ns – no statistical significance. (B) CD20 (B-lymphocyte marker) immunohistochemistry shows no significant difference in infiltrate cell counts between c-MYC+ and c-MYC- tumours; quantification shown in bar graph on right; Mean ± SEM; n = 11 for c-MYC+ and n = 9 for c-MYC- cohorts; ns – no statistical significance. (C, D) CD3 and F4-80 immunohistochemistry in NestinCre;c-MycSTOPFlox mice at different ages revealed no statistically significant increase in the number of T-lymphocyte (C) or macrophage (D) in the CP as compared to wild-type mice until tumour development at 9 months of age. Quantification bar graph on right side of C & D, Mean ± SEM; n for CD3 and F4-80 studies in time points P15 = 4, 3m = 3, 6m = 6, 9m = 4 for both mCP and mCPT cohorts; * P<0.05. Scale bar = 125 μm (A, B, C, D). (JPG 1187 kb) [file 40478_2019_739_MOESM7_ESM.jpg]

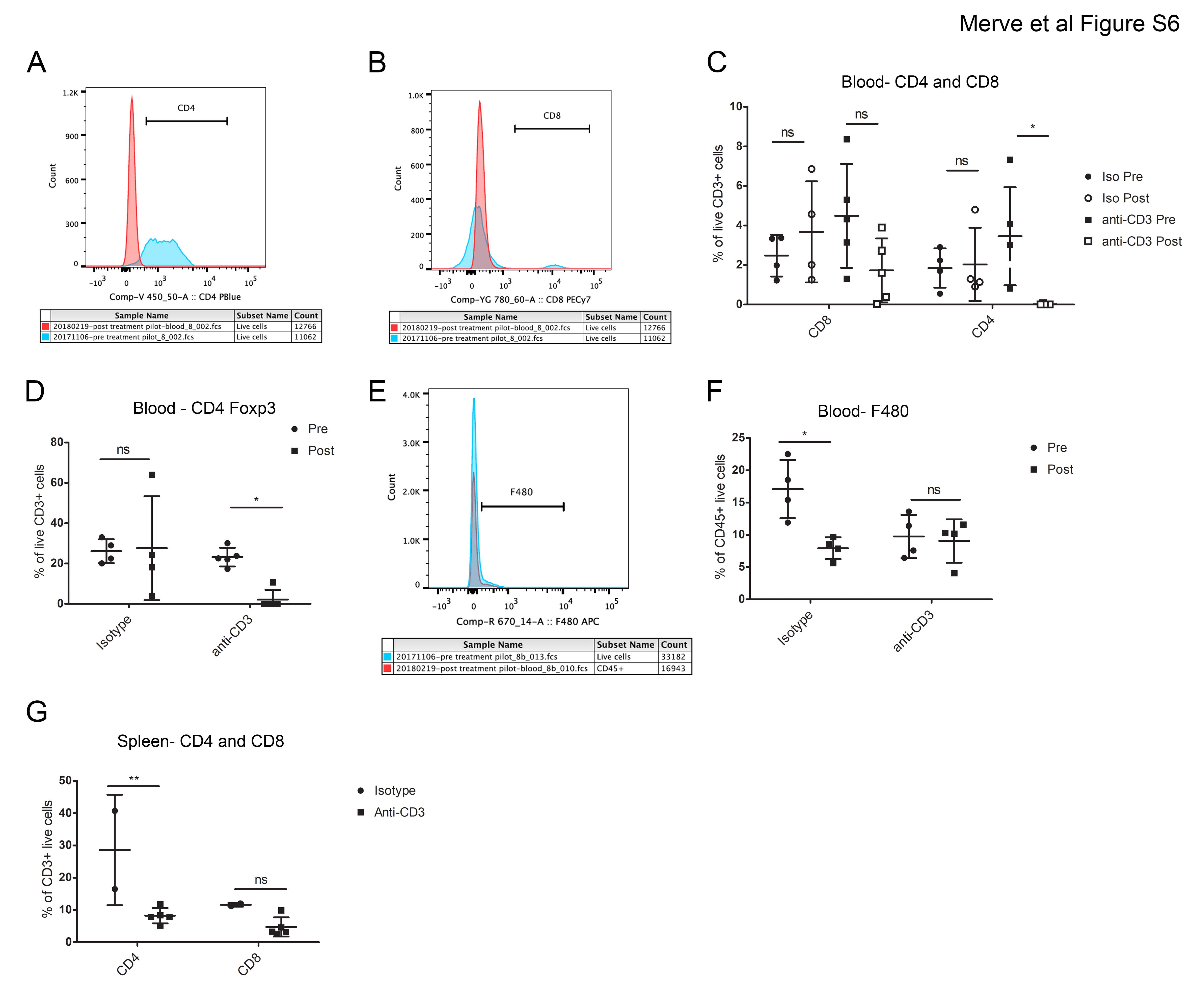

Supplement: Supplementary file 8 — Figure S6. Quantification of inflammatory cells in anti-CD3 treated experimental mice. (A-C) A significant reduction in CD4 but not CD8 subpopulation was noted in mouse blood following anti-CD3 treatment. Flow peaks showing a significant difference (red = post treatment, blue = pre-treatment) for CD4 (A) but not statistically significant difference for CD8 (B), which are quantified in the graph in (C) showing mean values ± SEM, n = 5 for isotype and n = 4 for anti-CD3 treated cohort, * P<0.05. (D) Reduction in CD4+ FoxP3+ (regulatory T-cells) population among CD3 T-cell population was noted following anti-CD3 injection, graph showing mean values ± SEM, n = 5 for isotype and n = 4 for anti-CD3 treated cohort, * P<0.05. (E, F) Anti-CD3 treatment had no effect on macrophage (F4-80) population as shown on flow peak (E) and graph in (F) showing mean values ± SEM, n = 5 for isotype and n = 4 for anti-CD3 treated cohort, * P<0.05. A reduction in F4-80 population was noted in post injection isotype treated group. (G) Reduction of mainly CD4, but not CD8 subpopulation is also seen in spleen; graph showing mean values ± SEM, n = 5 for isotype and n = 4 for anti-CD3 treated cohort, ** P<0.01. (JPG 659 kb) [file 40478_2019_739_MOESM8_ESM.jpg]
