# Supplementary material for: c-MYC overexpression induces choroid plexus papillomas through a T-cell mediated inflammatory mechanism
Source: Acta Neuropathol Commun. 2019 May 29;7:2. doi: 10.1186/s40478-019-0739-x (PMC6540455; doi:10.1186/s40478-019-0739-x)
Supplement: Supplementary file 2 — Table S1. Characteristics of CPT developing in the transgenic mouse model. Each tumour was histologically classified according to stratification of cells, loss of papillary architecture/solid growth and cellular & nuclear pleomorphism (- mild, moderate or severe). For mitotic activity mild = <2/10 HPF; moderate = 2 to 5/10HPF; severe = >5/10 HPF. Based on the WHO criteria the tumours were classified as either CPP (with mild features) or ACPP (with moderate features). None of the tumours fulfilled the criteria to be classified as CPC. Abbreviations: Het – heterozygous; Hom – heterozygous; Symp – symptomatic; End – end of experiment following 20, 5-6 or 9-12 months of observation (as applicable); F – female; M – male; FV – fourth ventricle; LV – lateral ventricle; CPP – choroid plexus papilloma; ACPP – atypical choroid plexus papilloma; Mod – moderate; Sev – severe. * All mice are of genotype RosaMyc;c-MycSTOPFlox. (DOCX 27 kb) [file 40478_2019_739_MOESM2_ESM.docx]

| N | Internal Histo Ref no. | Gentotype* (Het/Hom) | Age at collection (months) | Reason to terminate | Gender | Tumour Location | Other features | Histological Features | | | | | | Histological Diagnosis |
| --- | --- | --- | --- | --- | --- | --- | --- | --- | --- | --- | --- | --- | --- | --- |
|  |  |  |  |  |  |  |  | **Stratification of cells** | **Loss of papillary architecture/solid growth** | **Cellular and nuclear pleomorphism** | **Mitotic activity** | **Necrosis** | **Brain invasion** |  |
| 1 | SM12_122 | Het | 18 | Symp | F | FV |  | Mod | Mod | Mod | Mod | No | Yes | ACPP |
| 2 | SM12_125 | Het | 20 | End | F | LV |  | Mild | Mild | Mild | Mild | No | No | CPP |
| 3 | SM12_126 | Het | 20 | End | F | FV |  | Mild | Mild | Mild | Mild | No | No | CPP |
| 4 | SM12_127 | Het | 20 | End | M | LV & FV |  | Mild | Mild | Mild | Mild | No | No | CPP |
| 5 | SM12_128 | Het | 19 | Symp | F | LV & FV |  | Mild | Mild | Mild | Mild | No | No | CPP |
| 6 | SM12_129 | Het | 20 | End | F | LV & FV |  | Mild | Mild | Mild | Mild | No | No | CPP |
| 7 | SM12_130 | Het | 20 | End | F | FV |  | Mild | Mild | Mild | Mild | No | No | CPP |
| 8 | SM12_131 | Het | 20 | End | F | FV |  | Mild | Mod | Mild | Mild | No | No | CPP |
| 9 | SM12_139 | Hom | 11 | Symp | F | LV | HC | Mod | Mod | Mod | Sev | No | Yes | ACPP |
| 10 | SM12_142 | Het | 20 | End | M | LV & FV |  | Mild | Mild | Mild | Mild | No | No | CPP |
| 11 | SM12_143 | Het | 20 | End | F | LV (CPP), FV (ACPP) |  | Mod | Mild | Mild | Mild | No | Yes | ACPP |
| 12 | SM12_144 | Het | 20 | End | F | FV |  | Mild | Mild | Mild | Mild | No | No | CPP |
| 13 | SM12_145 | Het | 17 | Symp | F | LV & FV |  | Mod | Mod | Mod | Mod | No | Yes | ACPP |
| 14 | SM12_153 | Het | 20 | End | F | LV & FV | HC & DC | Mild | Mild | Mild | Mild | No | No | CPP |
| 15 | SM12_157 | Het | 16 | Dead | F | LV |  | Mild | Mild | Mild | Mild | No | No | CPP |
| 16 | SM12_160 | Het | 20 | End | F | LV & FV |  | Mild | Mild | Mild | Mild | No | No | CPP |
| 17 | SM12_161 | Het | 20 | End | F | LV & FV |  | Mild | Mild | Mild | Mild | No | Yes | CPP |
| 18 | SM12_162 | Het | 19 | Symp | M | LV & FV |  | Mild | Mild | Mild | Mild | No | No | CPP |
| 19 | SM13_12 | Het | 20 | End | F | LV & FV |  | Mild | Mild | Mild | Mild | No | No | CPP |
| 20 | SM13_13 | Het | 20 | End | F | LV & FV |  | Mild | Mild | Mild | Mild | No | No | CPP |
| 21 | SM13_15 | Het | 20 | End | M | LV & FV |  | Mild | Mild | Mild | Mild | No | No | CPP |
| 22 | SM13_16 | Het | 20 | End | M | LV & FV |  | Mild | Mild | Mild | Mild | No | No | CPP |
| 23 | SM13_17 | Het | 20 | End | F | LV & FV |  | Mild | Mild | Mild | Mild | No | No | CPP |
| 24 | SM13_18 | Het | 20 | End | F | LV & FV | HC | Mod | Mod | Mild | Mild | No | Yes | ACPP |
| 25 | SM13_20 | Het | 20 | End | M | LV & FV |  | Mild | Mild | Mod | Mild | No | No | CPP |
| 26 | SM13_22 | Het | 20 | End | F | LV & FV | HC | Mild | Mild | Mild | Mild | No | No | CPP |
| 27 | SM13_23 | Het | 20 | End | F | LV & FV |  | Mild | Mild | Mild | Mild | No | No | CPP |
| 28 | SM13_24 | Het | 20 | End | F | LV & FV |  | Mild | Mild | Mild | Mild | No | No | CPP |
| 29 | SM13_25 | Het | 20 | End | M | FV |  | Mild | Mild | Mild | Mild | No | No | CPP |
| 30 | SM13_26 | Het | 20 | End | M | FV |  | Mild | Mild | Mild | Mild | No | No | CPP |
| 31 | SM13_27 | Hom | 20 | End | M | LV & FV | HC | Mild | Mild | Mild | Mild | No | No | CPP |
| 32 | SM13_28 | Hom | 20 | End | F | FV | HC | Mod | Mod | Mod | Mild | No | No | ACPP |
| Developmental time points (collected before 20 months) | | | | | | | |  |  |  |  |  |  |  |
| 33 | SM17_40 | Het | 5 | End | M | FV |  | Mild | Mild | Mild | Mild | No | No | CPP |
| 34 | SM17_49 | Het | 5 | End | M | FV |  | Mild | Mild | Mild | Mild | No | No | CPP |
| 35 | SM18_32 | Hom | 6 | End | M | LV & FV | HC | Mild | Mild | Mild | Mild | No | No | CPP |
| 36 | SM17_38 | Hom | 9 | End | F | FV |  | Mild | Mild | Mild | Mild | No | No | CPP |
| 37 | SM17_90 | Het | 12 | End | M | LV & FV |  | Mild | Mild | Mild | Mild | No | No | CPP |
| 38 | SM17_111 | Het | 12 | End | M | LV & FV |  | Mild | Mild | Mild | Mild | No | No | CPP |
