# Supplementary material for: c-MYC overexpression induces choroid plexus papillomas through a T-cell mediated inflammatory mechanism
Source: Acta Neuropathol Commun. 2019 May 29;7:2. doi: 10.1186/s40478-019-0739-x (PMC6540455; doi:10.1186/s40478-019-0739-x)
Supplement: Supplementary file 4 — Table S2. Patient demographics and tumour characteristics. Paediatric and adult patients were included and the tumours samples were of all three histological grades (CPP, ACPP and CPC). Abbreviations: F – female; M – male; FV – fourth ventricle; LV – lateral ventricle; TV – third ventricle; met – metastasis; CPP – choroid plexus papilloma; ACPP – atypical choroid plexus papilloma; IHC – immunohistochemistry. For recurrence, Y = yes; for c-MYC expression, F = focal (10-50%); D = diffuse (>50%). * & ** are tumour tissue from same respective patients. *** is an autopsy case. (DOCX 22 kb) [file 40478_2019_739_MOESM4_ESM.docx]

| Tissue No. | Patient No. | Age | Gender | Site of tumour | Histology | Sample from recurrence? | C-MYC IHC |
| --- | --- | --- | --- | --- | --- | --- | --- |
| 1 | 1 | 34y | M | LV | CPC | Y (CPC recurrence, time not known) | 1 (F) |
| 2 | 2 | 10m | M | LV | CPC |  | 0 |
| 3 | 3 | 1y 11m | F | LV | CPC |  | 0 |
| 4* | 4 | 18y | M | Not known | CPC |  | 1 (F) |
| 5* | 4 |  |  |  | CPC | Y (CPC recurrence of (4) after 1 year) | 1 (F) |
| 6 | 5 | 1y 3m | F | LV | CPC |  | 1 (D) |
| 7 | 6 | 33y | F | Not known | ACPP |  | 0 |
| 8 | 7 | 60y | F | FV | ACPP | Y (after 13 years) | 0 |
| 9 | 8 | 2m | F | LV | ACPP |  | 0 |
| 10 | 9 | 60y | M | FV | ACPP |  | 0 |
| 11 | 10 | 52y | F | TV | ACPP | Y (recurrence after 6 years, 1st diagnosis - CPP in FV) | 1 (F) |
| 12 | 11 | 48y | F | FV | ACPP |  | 1 (D) |
| 13** | 12 | 50y | F | FV | CPP |  | 1 (F) |
| 14** | 12 | 53y | F | Spinal (met) | ACPP | Y Metastatic recurrence of (13) after 3 years. | 1 (F) |
| 15 | 13 | 5m | M | LV | ACPP |  | 1 (F) |
| 16 | 14 | 1y 8m | F | LV | ACPP |  | 1 (F) |
| 17 | 15 | 3y 1m | F | FV | ACPP |  | 1 (F) |
| 18 | 16 | 1y 10m | F | LV | ACPP |  | 0 |
| 19 | 17 | 5m | F | LV | ACPP |  | 0 |
| 20 | 18 | 37y | M | Not known | CPP |  | 0 |
| 21 | 19 | 9m | F | Not known | CPP |  | 1 (F) |
| 22 | 20 | 5m | M | LV | CPP |  | 0 |
| 23 | 21 | 2y | M | Not known | CPP |  | 0 |
| 24 | 22 | 3y | F | LV | CPP |  | 1 (F) |
| 25 | 23 | 5m | F | TV | CPP |  | 1 (F) |
| 26 | 24 | 3y | M | FV | CPP |  | 0 |
| 27 | 25 | 4m | M | Not known | CPP |  | 0 |
| 28 | 26 | 24y | M | FV | CPP |  | 1 (F) |
| 29 | 27 | 31y | M | LV | CPP | Y (after 8 years) | 1 (F) |
| 30 | 28 | 32y | M | FV | CPP |  | 0 |
| 31 | 29 | 50y | M | FV | CPP |  | 0 |
| 32 | 30 | 7m | F | Not known | CPP |  | 0 |
| 33 | 31 | 36y | F | FV | CPP |  | 0 |
| 34 | 32 | 13y | F | FV | CPP |  | 1 (F) |
| 35 | 33 | 23y | F | Not known | CPP |  | 0 |
| 36 | 34 | 9y | M | TV | CPP |  | 0 |
| 37 | 35 | 24y | M | LV | CPP |  | 0 |
| 38 | 36 | 75y | F | FV | CPP | Y (after 7 years) | 0 |
| 39 | 37 | 3y 2m | M | LV | CPP |  | 1 (F) |
| 40 | 38 | 1m | F | LV | CPP |  | 0 |
| 41 | 39 | 4y 10m | F | LV | CPP |  | 0 |
| 42*** | 40 | 42y | F | Spinal (met) | CPP | Y (primary 8 years ago, no other details available) | 0 |
